# New opportunities for creating quantum states of light and matter with intense laser fields

**DOI:** 10.1515/nanoph-2024-0605

**Published:** 2025-03-31

**Authors:** Nicholas Rivera

**Affiliations:** School of Applied and Engineering Physics, Cornell University, Ithaca, NY 14853, USA; Department of Physics, 1812 Harvard University, Cambridge, MA 02139, USA

**Keywords:** quantum optics, phononics, laser physics, high harmonic generation, nonlinear optics, squeezed states

## Abstract

Nonlinear dynamics provide an indispensable resource for creating quantum states of light, as well as other bosonic systems. Seminal work using second- and third-order nonlinear optical crystals, cavity quantum electrodynamics, and superconducting circuits, have enabled generating squeezed states, as well as various non-Gaussian quantum states (e.g., single photons, cat states) at both infrared and microwave frequencies. Nevertheless, it remains challenging to generate quantum states of light in broad portions of the electromagnetic spectrum: for example, at terahertz frequencies and at ultraviolet and X-ray frequencies. In this Perspective, I discuss a variety of emerging material platforms, as well as emerging theoretical and experimental tools, which enable overcoming these challenges. The main argument of this Perspective is that advances in driving nonlinear dynamics of material excitations, will enable generating quantum states of these material excitations as well as quantum states of light at new frequency ranges. I will further argue that in order to realize much of the promise of this nascent field, there is a need for innovation in the laser systems used to drive these nonlinear dynamics: specifically, innovations in realizing high-power laser sources that have very low noise, having quantum statistics similar to coherent states of light which describe lower intensity laser systems. I will highlight some experimental and theoretical work, in understanding quantum noise dynamics in complex laser systems, that can address these challenges.

## 1 Introduction

In the physical world, nonlinear systems offer an enormous range of rich and complex effects, many with wide-ranging implications in fundamental science and technology. The vast majority of the work on nonlinear systems is based on their description according to the laws of classical physics. At the same time, it has become increasingly important to understand the fundamental description of nonlinear systems according to the laws of quantum mechanics.

In part, this is because nonlinear dynamics leads to useful transformations of quantum fluctuations, the fundamental randomness of system states imposed by Heisenberg’s uncertainty principle. For example, nonlinearity generically enables generating states with non-classical fluctuation properties, such as squeezed states and entangled states [1], which may enable overcoming noise-related limits on a wide variety of important systems (e.g., interferometers [2], [3] imaging systems [4], [5], communication systems [6], [7], light sources such as lasers and frequency combs [8], [9], [10], [11], atomic clocks [12], [13], [14], magnetometers [15], and other emerging sensors [16], [17]).

Historically, the interplay between classical nonlinearity and quantum noise was first experimentally explored in the field of nonlinear optics [18], [19], [20], in nonlinear crystals and in optical fibers. While this interplay is still very actively explored in optics, it is now consequential for a much wider variety of physical platforms including optomechanics [21], spins [22], phonons [23], [24], [25], [26], [27], [28], [29], [30], [31], excitons [32], [33], [34], and Josephson junctions [35]. In each of these platforms, it is not quantum states of *light* that are created by nonlinearity, but quantum states of *matter*.

Realizing nonlinearities in new regimes will provide us with many new opportunities to generate quantum states of both light and matter with unique properties. That is the main argument of this Perspective. I will start by reviewing a universal theoretical connection between classical nonlinear dynamics and quantum effects such as squeezing and entanglement. This theory shows that the potential of a system to generate squeezing and entanglement is *completely* dictated by the *classical* nonlinear equations of motion describing the system.

Then, I will present a few new emerging platforms for realizing nonlinearities: optical phonons in polar insulators and semiconductors, spin waves in ferro- and antiferromagnets, and gases/solids irradiated by non-perturbative infrared driving fields. I will review the progress in realizing classical nonlinearities in these systems. In cases where efforts have been made to generate quantum states using these nonlinearities, I will discuss that prior art, and outline a few important remaining directions of inquiry. Otherwise, I will provide a plausibility argument for why quantum states should be realizable in a given driven material platform. In all cases, nonlinearities in these systems are accessed by driving these material platforms with strong laser fields.

I will argue that to realize many of the goals described above, there will be new required developments in laser physics itself: specifically in producing light sources that are simultaneously very intense, while having noise which is as low as possible – and sometimes even quantum levels of noise. I will outline some recent work in understanding quantum noise dynamics in complex laser systems, and highlight some of the major open theoretical and experimental questions that remain there.

It is important to mention that in a short Perspective such as this one, important topics will surely be left out. Examples of important concepts that I will not discuss here include nonlinearities in excitonic systems, superconducting circuits, optomechanics, integrated quantum photonics, and many others. Additionally, while I will focus a lot on squeezed states, the discussion applies equally well to the creation of entangled pairs of quanta and other multimode entangled states. I will not go in depth into the much richer (albeit harder to create and maintain) family of non-Gaussian quantum states important for quantum information science, such as single and multi-photon Fock states, Schrodinger cat and GKP states, and so on. Although I will not discuss these non-Gaussian states in depth, the creation of squeezed and entangled states by nonlinearities is a necessary and important precursor to these more complex nonclassical states. In particular, in many cases, the same interactions that lead to Gaussian squeezed and entangled states, when made stronger, produce useful non-Gaussian states (see e.g., [1]).

## 2 A general connection between nonlinear dynamics and quantum state generation

In this section, we elucidate a general connection between classical nonlinear dynamics and quantum squeezing effects. We start by briefly reviewing some of the simplest and arguably best-known models of squeezing effects. We then present a general framework for analyzing squeezing in more complex driven nonlinear systems. In particular we consider multimode nonlinear systems driven by light, where the driving light is not necessarily with light in coherent states, which we shall argue is crucial (in the Section 5).

We start by defining two concepts which are critical to this Perspective: nonlinearity, and modes. We start by defining nonlinearity for a generic wave. Suppose that we have a generic wave system described by a single classical wave field $\psi \left(x\right)$ , with *x* denoting spatial position (vector and tensor indices are suppressed for simplicity). This wave-field could represent light waves, lattice distortions in a solid (acoustic or optical), spin waves, water waves, etc. The discussion below is essentially unaltered by considering quantum field operators instead of classical fields. In a conservative system (i.e., without damping), the dynamics of the system are described by a Hamiltonian. A linear system corresponds to a quadratic Hamiltonian, i.e., one in which *ψ* appears at only linear or quadratic order (overall constants are unimportant). An example of a linear Hamiltonian would be $\int \text{d}x\frac{1}{2}\alpha {\left(\nabla \psi \right)}^{2}+\frac{1}{2}\beta \psi^{2}-F\left(x\right)\psi$ , with *α*, *β* being constants and $F\left(x\right)$ being a force. To connect this definition of linearity to the concept of linear response, we need only construct an equation of motion for *ψ*, which in this case would be: $\partial_{t}^{2}\psi -\alpha \nabla^{2}\psi +\beta \psi =F\left(x\right)$ (obtained by constructing the associated Lagrangian and using the Euler–Lagrange equations). In this case, the dependence of *ψ* on the force *F* is linear in *F*, since the homogeneous part of this differential equation is linear in *ψ*. Terms of cubic and higher-order in *ψ* would correspond to nonlinear terms in the equation. An example of a nonlinear Hamiltonian would be

(1) $$H_{NL}=\int \text{d}x\frac{1}{2}\alpha {\left(\nabla \psi \right)}^{2}+\frac{1}{2}\beta \psi^{2}-F\left(x\right)\psi +V_{NL}\left(\psi \right),$$

with $V_{NL}\left(\psi \right)=\frac{1}{3}c_{3}\psi^{3}+\frac{1}{4}c_{4}\psi^{4}+\ldots$ being the nonlinear potential, where the ellipses denote higher-order terms.

In the discussion so far, we have considered a single field. For multiple fields, $\left(\psi_{1},\psi_{2},\ldots \right)$ , the linearity condition is that the Hamiltonian is again a degree-two polynomial at most (so no cubic terms like $\psi_{1}^{2}\psi_{2}$ or higher-order). A single nonlinear term (cubic or above) renders the entire system behavior nonlinear, even if one of the fields appears in at-most-quadratic-order in the Hamiltonian. In the literature, nonlinearities are occasionally divided into perturbative and non-perturbative nonlinearities. In the perturbative case, the nonlinear potential *V*_*NL*_(*ψ*) can be truncated at some order in *ψ* (e.g., third or fourth). We will visit an example of perturbative nonlinearities for phonons in Section 3 on phonons and magnons. In the non-perturbative case, relevant to high-harmonic generation to be discussed in Section 4, the nonlinear potential is a more general function of *ψ* where the physics cannot be described in terms of a low-order truncation of the nonlinear potential.

Another key concept to describe is that of a mode. While this concept has some subtlety, especially in nonlinear as well as open (dissipative) systems, we will keep the discussion simple by confining ourselves to two important and highly relevant limits. In particular, we consider the case in which damping is not so large such that we can refer to a complete set of orthonormal modes. Further, we consider the case in which nonlinearity is not so large that the modes of the underlying linear system are a useful way to describe the system dynamics. Therefore, we can write our wavefield as a sum over orthonormal modes. For a real wave field *ψ*, we can write $\psi \left(x\right)=\sum_{m}\alpha_{m}u_{m}\left(x\right)+\alpha_{m}^{*}u_{m}^{*}\left(x\right)$ , where the $u_{m}\left(x\right)$ are eigenfunctions of the linear wave equation, and the *α*_*m*_ are expansion coefficients similar to Fourier coefficients. In the case of quantized bosonic fields, the $\alpha_{m},\alpha_{m}^{*}$ are “promoted” to annihilation and creation operators $a_{m},a_{m}^{†}$ , satisfying canonical commutation relations $\left[a_{m},a_{n}^{†}\right]=\delta_{mn}$ . Going back to the discussion on nonlinearity in the previous paragraphs, nonlinearity in the language of creation and annihilation operator corresponds to the presence of terms which are higher-than-quadratic order in the *a* and *a* ^†^ operators (for example, the term *a* ^†^ *a* ^2^ is a nonlinear term in the Hamiltonian while *a* ^2^ is not). The physics of squeezing and quantum noise more broadly is enriched by the presence of multiple modes. For example, quantum states such as single-mode squeezed states are states with reduced noise of some observable in a *single* mode, while entangled states are those with non-classical correlations between *multiple* modes. Squeezing versus entanglement will be elucidated later in this section.

We now review a few of the best-known models of squeezing in optics. In nonlinear optics, there are a variety of platforms for generating squeezed light. Squeezed light can roughly be categorized into squeezed vacuum (with zero average electric field), and displaced-squeezed vacuum (with non-zero average field). Examples of platforms for generating squeezed light include second-order nonlinear media, atomic gases, and third-order nonlinear media (most famously optical fibers, but recently also integrated waveguides and cavities). Despite the variety of platforms, the most successful squeezing platform in terms of applications is second-order nonlinear media – in part due to the lower optical powers at which nonlinear squeezing effects can become efficient. Let us consider a simple case in which there is a strong coherent pump laser at frequency 2*ω* and a mode at frequency *ω* which is initially dark (no photons in it). In this case, where the pump can be treated classically, the effective Hamiltonian for the lower-frequency mode, reduces from cubic to quadratic, and is given in the interaction picture as:

(2) $$V/\hbar =ig\left(a^{2}-a^{†2}\right)\text{,}$$

with *g* being a constant and *a* being a harmonic oscillator annihilation operator [36]. The quantum state of this mode, $|\left.\psi \left(t\right)\right\rangle$ , with *t* being time, is then given from Eq. (2) via

(3) $$\left|\left.\psi \left(t\right)\right\rangle \right.={\text{e}}^{r\left(a^{2}e^{-2i\omega t}-a^{†2}e^{2i\omega t}\right)}\left.\left|0\;\right.\right\rangle ,$$

with *r* = 2*gt*, with *t* the interaction time. Equivalently to the state transformation of Eq. (3), we can express the squeezing in terms of transformations of the creation and annihilation operators. The annihilation operator at the output, denoted

(4) $$a_{\text{out}}=\mu \left(t\right)a_{\text{in}}+\nu \left(t\right)a_{\text{in}}^{†},$$

with $\mu \left(t\right)={\text{e}}^{-\text{i}\omega t}\cosh r,\nu \left(t\right)=-{\text{e}}^{-\text{i}\omega t}\sinh r$ . From this transformation, we may compute the variance of the “position” quadrature $X=a_{\text{out}}+a_{\text{out}}^{†}$ , ${\left(\Delta X\right)}^{2}=\langle X^{2}\rangle -{\left\langle X\right\rangle }^{2}$ and well as the variance of the “momentum” quadrature $P=i\left(a_{\text{out}}^{†}-a_{\text{out}}\right)$ , defined similarly. We consider the position quadrature for concreteness. From these transformations, we can see the variance of the position oscillates as:

(5) $${\left(\Delta X\right)}^{2}=\frac{1}{2}\left({\text{e}}^{2r}\left(1-\cos2\omega t\right)+{\text{e}}^{-2r}\left(1+\cos2\omega t\right)\right),$$

while the mean remains zero in time if the initial state is the vacuum state. Once the parametric interaction ends, the variance of the freely evolving mode oscillates at twice the frequency of the parametrically generated mode frequency, and oscillates between a maximum of e^2*r*^ and a minimum of e^−2*r*^ [36] (ignoring the time-dependence of *r* itself, which is valid since typically *ω* ≫ 2*g*). The state repesented equivalently by Eqs. (3) and (4), known as squeezed vacuum, can be produced by this interaction in optical parametric amplifiers and oscillators. Although the mean amplitude of the field is zero, the number of photons is not, going as $\langle n\left(t\right)\rangle =\sinh^{2}r$ .

It is important to point out that the model of squeezing presented above is an idealized one. In practice, when realizing squeezed states in experiments, there are several requirements that need to be met. One such requirement is *phase-matching*: in propagating-wave parametric amplifiers, one not only needs a field at half the frequency of the drive, but the wavevectors of the generated photons also need to be consistent with momentum conservation. When energy-momentum conservation (phase-matching) is satisfied, the parametrically generated field can resonantly build up. In practice, a powerful technique for aiding phase-matching is *poling*, which involves creating a spatial modulation of the optical properties (e.g., periodic along the propagation direction of light); this additional spatial dependence can strongly enhance the efficiency of the nonlinear interaction [37], establishing a so-called quasi-phase matching. Beyond having resonance, one also needs a sufficient length of interaction; in bulk second-order nonlinear media, this is not too stringent a requirement, but it can be enhanced for example by use of a cavity (creating a parametric oscillator instead). Beyond generating the squeezing, it is also important to preserve and detect the squeezing. Importantly, dissipation (e.g., attenuation, scattering, inefficient detection, etc.) “spoils” squeezing, because the dissipation mixes in uncorrelated vacuum fluctuations. In particular, suppose one has a squeezed state of light with *n* photons, and suppose it is attenuated such that after attenuation, the state has *ℓn* photons. Then the quadrature variance ${\left(\Delta X\right)}^{2}$ becomes

(6) $${\left(\Delta X\right)}^{2}\to \ell {\left(\Delta X\right)}^{2}+\left(1-\ell \right).$$

As $\ell \to 0,{\left(\Delta X\right)}^{2}\to 1$ corresponding to the vacuum level [38], and obscuring any squeezing which may have been present. Thus, in experiments where one wishes to detect squeezing, it is important to avoid large degrees of loss. This point is of major importance in the sections that follow, on generating squeezed states in condensed matter systems. The last important experimental point I mention here is that typically, squeezing is detected by interfering the squeezed light with a coherent state of much higher intensity, called the local oscillator [38]. In particular, what is common is a type of balanced homodyne detection, in which the local oscillator and squeezed light are combined on a 50/50 beamsplitter, and then sent to two photodiodes, whose photocurrents are then subtracted. The signal is then proportional to the product of the local oscillator field and the squeezed light field (the latter of which has zero mean and fluctuates). The presence of the strong coherent state amplifies the quantum fluctuations of the squeezed state to a point at which it is readily discernable in the Fourier transform of the electric current produced by a photodiode (yielding a noise power spectral density). In order for this procedure to work, the local oscillator has to be able to interfere effectively with the squeezed state, which happens for example if the two are in the same mode. This is especially relevant for pulsed squeezed states and other multimode squeezed states [39]. We mention that while we have discussed much of these ideas in the photonics context, similar ideas apply in other bosonic systems where squeezing has been generated such as optomechanics and excitonics (see Section 1 for relevant references).

We should also discuss the case of squeezing via third-order nonlinearities and the Kerr effect, which while long-studied, has been less explored than the second-order nonlinear case. In part, this is because accessing nonlinearities via the Kerr effect has traditionally required much higher optical powers, being efficient only for pulsed light or cavities. Further the often-high powers needed to achieve Kerr-squeezing can cause other deleterious processes to be efficient (e.g., multiphoton absorption, harmonic generation, coupling to material degrees of freedom a la the Raman effect, etc.). Nevertheless, the major advantage of Kerr-squeezing approaches is that they are realizable in any material (all materials have third-order nonlinearity, unlike the second-order case, which requires centro-symmetry breaking). Further, phase matching is un-necessary in many cases phase matching is easy to satisfy in many cases due to similar frequencies of the interacting modes. And, in high-power laser systems, Kerr effects can be efficiently accessed.

As in the second-order nonlinear case, we consider the simplest model possible: a single-mode undergoing self-phase modulation (the Kerr effect). The relevant interaction Hamiltonian is [36]

(7) $$H/\hbar =\omega a^{†}a+\frac{K}{2}a^{†2}a^{2}.$$

Although the Hamiltonian of Eq. (7) can be exactly exponentiated, it will be instructive to make an approximation. Since any nonlinear effect needs a strong driving field to become efficient, we can say that

(8) $$a\left(t\right)=\alpha \left(t\right)+\delta a\left(t\right),$$

where $\alpha \left(t\right)=\left\langle \psi \left|a\left(t\right)\right|\psi \right\rangle$ is the mean *c*-number amplitude (with $|\left.\psi \right\rangle$ the initial system state), and *δa*(*t*) is a fluctuation operator. The statistical properties of *a* are encoded in *δa*. In essentially all cases where nonlinearities are triggered by many photons, the fluctuation operators can be taken as small compared to the mean, leading to Eq. (8) being a useful separation. In this approximation, the operator equations of motion (Heisenberg equations) reduce to linear equations for the fluctuation operators, which will imply Gaussian statistics for the output light. The equations of motion for the fluctuation operators are then

(9) $$\left(\text{d}/\text{d}t\right)\delta a=-i\omega \delta a-iK\left(2{\left|\alpha \left(t\right)\right|}^{2}\delta a+\alpha {\left(t\right)}^{2}\delta a^{†}\right),$$

where $\alpha \left(t\right)={\text{e}}^{-\text{i}\left(\omega +K{\left|\alpha \left(0\right)\right|}^{2}\right)t}\alpha \left(0\right)$ , and the equation for *δa* ^†^ follows by Hermitian conjugation. While we won’t give the exact analytical solution here (see [1]), the important thing is that we can write the solution as a Bogoliubov transformation of the form $a_{\text{out}}=\mu_{K}\left(t\right)a_{\text{in}}+\nu_{K}\left(t\right)a_{\text{in}}^{†}$ , similar to Eq. (4). This transformation automatically entails squeezing, and also enforces that the Wigner distribution of the output light, which is a phase-space distribution, is Gaussian. This is the sense in which the linearization approximation enforces Gaussian statistics.

Before moving on to a more general treatment of squeezing, we point out that the discussion of squeezing above has primarily been focused on *single-mode* squeezing, defined by a Bogoliubov transformation of the form: *a*′ = *α* + *μa* + *νa* ^†^ (where *α* is a c-number corresponding to a displacement). More generally, nonlinearities can cause quantum correlations between two or more modes, leading to a *multimode squeezed state*. There, the relevant Bogoliubov transformation is of the form:

(10) $$a_{i}=\alpha_{i}+\underset{j}{\sum }\mu_{ij}a_{j}+\nu_{ij}a_{j}^{†},$$

where the index *i* labels different modes (which for light could be frequency bins of a pulse, cavity modes of a multimode resonator, spatial modes in a light beam, polarization modes, etc.). In the case of two-mode squeezing, nonclassicality manifests through a reduced variance relative to multimode coherent states, in observables such as sums and differences of quadratures of the two modes of interest. In the multimode case, these quantum correlations between different modes can be seen also as multimode *entanglement*.

From the examples above, we see that squeezing generically occurs when creation and annihilation operators are coupled together. In what follows, I’ll show that this coupling occurs in essentially any classical nonlinear system (when quantized). To do that, I will review a powerful and general connection between classical nonlinear dynamics and squeezing effects. The connection was first introduced by us in earlier work, see e.g., Refs. [40], [41], and a self-contained derivation of the theory is given in the Supplementary Information (SI) of this work.

Consider a general nonlinear physical system, which can have both electromagnetic field and matter degrees of freedom. Electromagnetic degrees of freedom can refer to any type of solution to Maxwell’s equation: plane waves modes, cavity modes, temporal modes, etc. Matter degrees of freedom are also broadly defined: these can include a collection of inverted two-level systems (determining a gain medium), phonons in a fiber or waveguide, or particles described by position and momenta.

Defining the collection of independent degrees of freedom (and their conjugates) as ***α***, α* **,** we define the classical theory of the system as an input–output relation, relating the degrees of freedom at the final time, $\alpha_{\text{out}},\alpha_{\text{out}}^{*}$ , to those at the initial time $\alpha_{\text{in}},\alpha_{\text{in}}^{*}$ . In particular, we write $\alpha_{\text{out}}=F\left[\alpha_{\text{in}},\alpha_{\text{in}}^{*}\right]$ , where *F* is some arbitrary function of the light and matter variables. We will also refer to the components of the vector ***α***_out_ as *α*_*i*_.

In what follows, we consider the quantum noise dynamics of this system. We consider the most common case in which the quantum fluctuations are small compared to the mean values of the quantities (as we did in the case of the Kerr effect, described previously), permitting an expansion of the quantum dynamics in powers of the fluctuations $\delta a_{i},\delta a_{i}^{†}$ , such that *a*_*i*_ ≡ *α*_*i*_ + *δa*_*i*_, where $\alpha_{i}=\left\langle a_{i}\right\rangle$ . This is true in nearly all cases where nonlinearities are triggered by a number of photons (or other excitations) which is much larger than one. In this limit, we claim the following rule for determining the final quantum state of the system: associate the classical degrees of freedom $\alpha_{i},\alpha_{i}^{*}$ with bosonic annihilation and creation operators $a_{i},a_{i}^{†}$ . This can also be done for most matter degrees of freedom, in the limit where the number of matter particles is large in some characteristic spatial scale of variation of the system – in that case, the matter can be described in terms of bosonic fields.1This is the essence of what is today called macroscopic quantum electrodynamics. See reviews such as Refs. [96], [97] for more details. Then, the output operators $a_{i,\text{out}},a_{i,\text{out}}^{†}$ can be written in terms of the input operators $a_{i,\text{in}},a_{i,\text{in}}^{†}$ in the form of a multimode Bogoliubov transformation, like Eq. (10). In particular (see discussion around Eqs. S7–9 of the SI for description):

(11) $$\begin{array}{ll} a_{i,\text{out}} & =\alpha_{i,\text{out}}+\underset{j}{\sum }\mu_{ij}\delta a_{j,\text{in}}+\nu_{ij}\delta a_{j,\text{in}}^{†},\;\text{with}\;\\ \mu_{ij} & ={\left(\frac{\partial \alpha_{i,\text{out}}}{\partial \alpha_{j,\text{in}}}\right)}_{\alpha_{\text{in}},\alpha_{\text{in}}^{*}},\;\nu_{ij}={\left(\frac{\partial \alpha_{i,\text{out}}}{\partial \alpha_{j,\text{in}}^{*}}\right)}_{\alpha_{\text{in}},\alpha_{\text{in}}^{*}}. \end{array}$$

The term *α*_*i*,out_ is found by solving the classical nonlinear problem. The Bogoliubov coefficients *μ*_*ij*_ and *ν*_*ij*_ are the Jacobian matrix (or gradients) of the classical dynamics: they are the sensitivities of the classical final state: $\alpha_{\text{out}}=F\left[\alpha_{\text{in}},\alpha_{\text{in}}^{*}\right]$ , to changes in the classical initial conditions. We should mention that although it looks like this formalism only works for systems described by bosonic operators, that is not the case: for systems described by a position and velocity (like a particle in a potential), the quantities *α*, α* get replaced by *x*, *p* (the position and momentum), and noise is found by differentiating various observables with respect to the initial positions and momenta. Because of the connection of quantum noise and sensitivity to initial conditions, we refer to this framework as *quantum sensitivity analysis*.

As a quick example, let us evaluate the Bogoliubov transformation in the case of the Kerr effect. The classical equation of motion describing self-phase modulation of a single-cavity mode was given above as $\alpha_{\text{out}}={\text{e}}^{-\text{i}\left(\omega t+\theta \alpha_{\text{in}}^{*}\alpha_{\text{in}}\right)}\alpha_{\text{in}}$ . Here we have made the replacements: $\alpha \left(0\right),\alpha \left(t\right)\to \alpha_{\text{in}},\alpha_{\text{out}}$ , and *Kt* → *θ*. From the rule above, we immediately find: $\mu ={\text{e}}^{-\text{i}\left(\omega t+\theta {\left|\alpha_{\text{in}}\right|}^{2}\right)}\times \left(1-i\theta {\left|\alpha_{\text{in}}\right|}^{2}\right)$ and $\nu ={\text{e}}^{-\text{i}\left(\omega t+\theta {\left|\alpha_{\text{in}}\right|}^{2}\right)}\times \left(-i\theta \alpha_{\text{in}}^{2}\right)$ . To see this, we need to compute $\mu =\frac{\partial \alpha_{\text{out}}}{\partial \alpha_{\text{in}}}$ and $\nu =\frac{\partial \alpha_{\text{out}}}{\partial \alpha_{\text{in}}^{*}}$ (since there is only a single mode, the mode labels *i*, *j* are omitted). Note further that *α*_in_ and $\alpha_{\text{in}}^{*}$ are independent variables. The resulting expressions for *μ*, *ν* are in perfect agreement with the standard approach based on the linearization of the operator Heisenberg equations [1]. Notice of course that in the absence of nonlinearity, *μ* = 1, *ν* = 0 as expected. The transformation also respects ${\left|\mu \right|}^{2}-{\left|\nu \right|}^{2}=1$ as expected (the latter is needed to preserve the commutation relations of the output operators).

The same quantum sensitivity analysis framework also prescribes the variance (noise) of arbitrary observables. For example, let us consider the variance (or noise) of an arbitrary quantity *X* *.* *X* could be intensity in one or more modes, phase, quadrature, etc. Classically, *X* is a function of the initial conditions of the system, i.e., the initial amplitudes of different light modes and the initial values of degrees of freedom of different matter excitations. Denoting the collection of all initial amplitudes as $\alpha_{\text{in}},\alpha_{\text{in}}^{*}$ , we can say $X_{\text{out}}=X_{\text{out}}\left(\alpha_{\text{in}},\alpha_{\text{in}}^{*}\right)$ . The theory developed in Refs. [40], [41] then prescribes the variance of *X* as:

(12) $$\begin{array}{ll} {\left(\Delta X_{\text{out}}\right)}^{2} & =\left(\partial X_{\text{out}}/\partial \alpha_{\text{in}}\partial X_{\text{out}}/\partial \alpha_{\text{in}}^{*}\right)\\ & \;\times {\left(\begin{array}{cc} \left\langle \delta a\delta a\right\rangle & \left\langle \delta a\delta a^{†}\right\rangle \\ \left\langle \delta a^{†}\delta a\right\rangle & \left\langle \delta a^{†}\delta a^{†}\right\rangle \end{array}\right)}_{\text{in}}\left(\begin{array}{c} \partial X_{\text{out}}/\partial \alpha_{\text{in}}\\ \partial X_{\text{out}}/\partial \alpha_{\text{in}}^{*} \end{array}\right). \end{array}$$

The correlation matrix (with entries such as $\left\langle \delta a\delta a\right\rangle$ ) contains the quantum statistics of the initial fields (e.g., coherent, squeezed, thermal, multimode entangled). The new insight developed in [40], [41] lies in the derivatives ∂*X*_out_/∂ ***α***_in_: they are the change in *X* due to changing the classical initial conditions. Therefore, the dynamics of quantum fluctuations are completely prescribed by the classical dynamics and the statistics of the input light. In the case where the initial state is a multimode coherent state, and Eq. (12) simplifies to:

(13) $${\left(\Delta X_{\text{out}}\right)}^{2}=\underset{k}{\sum }{\left|\frac{\partial X_{\text{out}}}{\partial \alpha_{\text{in},k}\left(0\right)}\right|}^{2}={\left|\nabla_{\alpha_{\text{in}}}X_{\text{out}}\right|}^{2}.$$

It is worth pointing out that from these expressions above (Eqs. (12) and (13)), it is not obvious “what” quantities are squeezed for a given nonlinear interaction: in other words, which quantities *X* have a smaller variance than one would expect for a coherent state output. This however can be solved via the Bloch–Messiah decomposition known in the field of quantum information science, which expresses a given multimode quantum state in terms of a set of independent squeezing modes. In particular, due to the fact that *μμ* ^†^ − *νν* ^†^ = 1, where *μ*, *ν* are matrices of Bogoliubov coefficients, it is possible to perform a joint singular value decomposition on the *μ* and *ν* matrices: *μ* = *F*Σ_*μ*_ *G*, *ν* = *F*Σ_*ν*_ G*, where $F\left(G\right)$ define the output (input) “Schmidt modes”, which are certain linear combinations of the input operators in the original basis chosen [42]. Meanwhile, the diagonal matrices $\Sigma_{\mu }=\mathrm{diag}\left(\cosh r_{1}\cdots \cosh r_{N}\right),\Sigma_{\nu }=\mathrm{diag}\left(\sinh r_{1}\cdots \sinh r_{N}\right)$ encode the squeezing levels of the different principal modes (the squeezing level of mode *i* is ${\text{e}}^{2r_{i}}$ ). This decomposition can be seen as taking the input modes, performing a linear operation on them (*G*) which mixes the input modes, applying single-mode squeezing transformations to the different modes independently, and then applying another linear-mixing operation (*F*). Further, from these Bogoliubov matrices, we can also evaluate the entanglement of different modes, in analogy with the case of two-mode squeezing.

I stress that this framework is exactly equivalent in physical content to the main approach used to describe squeezing dynamics: linearization of the Heisenberg operator equations (as we did in the single-mode Kerr effect), followed by solution of these linearized equations [1], [43]. In the limit where quantum fluctuations are small compared to the mean classical fields, the framework presented here is also equivalent to a “stochastic equations” framework, where the classical equations are solved for an ensemble of initial conditions. The ensemble of initial conditions is often “drawn” from a multivariate Gaussian distribution whose mean and variance are prescribed by quantum mechanics [44] and accurately emulate the effect of quantum fluctuations. Despite the equivalence of these different approaches, the framework I present here, which is the newest, comes with important theoretical and computational advantages.2Theoretically, the quantum sensitivity analysis approach reveals a powerful design rule for minimizing quantum noise: minimize the sensitivity of some system observable to *all* possible changes in initial conditions, even sensitivities to modes which are not populated (but which still contribute vacuum fluctuations). It also reveals right away that a wide variety of nonlinear systems which have a tendency towards chaos will strongly amplify quantum noise. One of the most powerful theoretical and computational advantages of this framework comes from the *analytical* separation of the sensitivities and the initial correlations of the input. This means if the quantum statistics of the input changes, the noise can be immediately determined once one knows the relevant sensitivities. The role of the input quantum statistics is important when understanding how a system responds to squeezed light (which is important in sensing and interferometry) and also in understanding how excess noise in the input of a nonlinear system affects squeezing. This computational simplicity is in contrast to the other approaches (linearization and stochastic simulations), where a new simulation must be done to take into account a new noise distribution. In the case of linearization, it is in principle possible to capture a change in the noise distribution without doing a new simulation (i.e., without numerically solving a set of differential equation), if one computes a Green’s function for the linearized equations. However, computing a Green’s function in a highly multimode system (which can have thousands of degrees of freedom), is very computationally intensive, requiring essentially 2*N* simulations of the linearized equations, where *N* is the number of degrees of freedom. For quantum sensitivity analysis, if we have a specific observable we want to compute, then computational adjoint methods allow the noise to be computed using two simulations. Changing the input noise distribution requires no new simulations. By simulation, we mean numerically solving a set of *N* differential equations, where for many systems *N* ≫ 1. Importantly, because of the rise of computational adjoint methods in computational design and optimization, it is possible to compute the gradient $\nabla_{\alpha \left(0\right)}X$ using only *two* simulations (effectively a forward solve and a backward solve of the differential equation) [98], [99]. Similarly, for calculating the noise of specific observables, this approach outperforms stochastic simulations, where a large number of simulations needs to be done for an ensemble of initial conditions until statistical convergence is achieved (this can require thousands of simulations for sufficient convergence).


The major new insight which is manifest from the framework reviewed here [45] here is that the quantum noise dynamics of a system can be completely predicted from its underlying classical model: by taking derivatives of the classical dynamics. In this sense, the quantum sensitivity analysis framework reveals a “hidden” quantum dimension to classical systems: I mean this colloquially, in the sense that the classical theory “knows” about certain quantum statistical effects. Further, we see, from the Bogoliubov coefficients *μ*_*ij*_, *ν*_*ij*_ introduced above, that squeezing in some observables should be anticipated for a generic nonlinearity, since essentially any conceivable nonlinear term can couple a complex field to its conjugate.

It is important to emphasize at this stage that although we are predicting quantum statistics from “classical dynamics”, this does not limit the treatment to only predicting quantum statistics of *light.* Matter systems, and their quantum statistics, are also accessible from this framework, as many matter systems have a corresponding classical description as well. For example,3Let us consider the dynamics of a quantum particle (such as an electron) propagating in free space. A particle prepared in a wavepacket with some initial position and momentum spread *σ*_*x*_(0), *σ*_*p*_(0) will spread spatially (but not in momentum, since momentum is conserved). We now show that the rate of spreading is perfectly predicted by the quantum sensitivity analysis approach. The classical equations of motion are $\dot{x}=p/m$ and $\dot{p}=0$ , with *x* being position, *p* being momentum, and *m* being the particle mass. The solution is $x\left(t\right)=x\left(0\right)+p\left(0\right)t/m$ , and $p\left(t\right)=p\left(0\right)$ . Assuming an initial condition where *x* and *p* are uncorrelated (so that ⟨*xp* + *px*⟩ − 2⟨*x*⟩⟨*p*⟩ = 0), quantum sensitivity analysis yields for the final position uncertainty: $\sigma_{x}^{2}\left(t\right)={\left(\frac{\partial x\left(t\right)}{\partial x\left(0\right)}\right)}^{2}\sigma_{x}^{2}\left(0\right)+{\left(\frac{\partial x\left(t\right)}{\partial p\left(0\right)}\right)}^{2}\sigma_{p}^{2}\left(0\right)=\sigma_{x}^{2}\left(0\right)+\sigma_{p}^{2}\left(0\right)t^{2}/m^{2}$ . This follows from Eq. (S14) of the Supplementary Information. The momentum uncertainty also follows from Eq. (S14) as: $\sigma_{p}^{2}\left(t\right)={\left(\frac{\partial p\left(t\right)}{\partial x\left(0\right)}\right)}^{2}\sigma_{x}^{2}\left(0\right)+{\left(\frac{\partial p\left(t\right)}{\partial p\left(0\right)}\right)}^{2}\sigma_{p}^{2}\left(0\right)=\sigma_{p}^{2}\left(0\right)$ . Both of these are the correct result (see e.g., [100], and note that for a particle in a pure state, $\sigma_{p}\left(0\right)=\hbar /2\sigma_{x}\left(0\right)$ ). we show how this framework predicts one of the most basic phenomena in quantum mechanics, the spreading of the wavefunction of a free particle. In the SI, we show how our theory also describes the quantum noise added by a gain medium (which corresponds to an excited matter reservoir). While our framework cannot address situations where fluctuations diverge, such as the onset of phase transitions (such as Bose–Einstein condensation, superconductivity, etc.), our framework would correctly describe the excitations of such systems, which can be understood as fluctuations on top of an ordered state. For example, the dynamics of Bogoliubov excitations of a Bose condensate should be accurately described based on a quantum sensitivity analysis of the Gross–Pitaevskii equation [46].

This observation informs the “thesis” of this Perspective: *that emerging platforms for accessing nonlinear dynamics in materials should enable new sources of quantum light, even if the underlying nonlinearities are different in form from those that have been long-studied in optics (namely non-resonant second- and third-order nonlinearities).* This program, of identifying quantum effects in new classical nonlinear systems, can be made algorithmic from the standpoint of quantum sensitivity analysis. In particular: (1) take some nonlinear system, potentially with many degrees of freedom, for which we have a classical description, (2) analytically compute, or simulate, the dynamics along with their gradients, (3) compute noise/correlations for any input statistics, and (4) then use tools like Bloch-Messiah to find the degrees of freedom which are most strongly squeezed. This new framework should allow one to very efficiently find so-far hidden quantum effects (in potentially very complex) systems which haven’t been studied this way. We will give an example of this in the next section.

## 3 Quantum optics at terahertz frequencies based on driven collective excitations

In this section, we introduce two emerging platforms for nonlinearity that should enable generating quantum light at terahertz frequencies, a frequency range in which squeezing and entanglement has not yet been realized. We should note that in using the word terahertz, we are broadly including light of frequencies ranging from 1 THz to a few tens of THz; the upper part of this range is often referred to as mid-infrared. The importance of realizing squeezing and other quantum effects at these frequencies, while being of intrinsic fundamental interest, may also lead to important improvements in applications which make use of terahertz sources. For example, squeezing at terahertz frequencies could open up opportunities in molecular spectroscopy and terahertz communications. Recently, the response of materials to intense pulses of mid-infrared and terahertz light is coming into focus. In part, this is due to steady technological advancements in the efficiency of sources in this spectral range, which are typically realized based on difference-frequency generation processes that convert nearby frequencies of an IR-vis pulse into a broad spectrum of much lower-frequency radiation. Light fields at these frequencies can resonantly drive motion of the lattice (phonons) of a material, as well as motion of spins (magnons).

Let us start with the case of phonons. Phonons can be categorized as acoustic or optical. At zero wavevector (a good approximation for the wavevector of the driving light, due to the fact that the wavevector of light is much smaller than a reciprocal lattice vector of the underlying crystal), optical phonons have a finite frequency while the acoustic phonons are at zero frequency. Hence, light drives optical phonons, provided that they have a finite electric dipole motion associated with their oscillation. Such dipole-carrying optical phonons are called infrared-active, as opposed to Raman-active phonons, which can still be excited by light, albeit much less efficiently.

The response of an infrared-optical phonon to light can be understood most simply in terms of a simple classical model of a driven anharmonic oscillator. For a particular optical phonon mode, we can define a mode amplitude for the *i* phonon mode, denoted *Q*_*i*_ with dimensions of ${\left(\text{mass}\right)}^{1/2}\times \text{length}$ – as is customary in the field of *coherent phononics* – which satisfies an equation of motion of the form [45], [47] :

(14) $$\frac{{\text{d}}^{2}}{\text{d}t^{2}}Q_{i}+\gamma \frac{\text{d}}{\text{d}t}Q_{i}+\omega_{0}^{2}Q_{i}+V_{NL}^{\prime }\left(\left\{Q\right\}\right)=Z_{i}^{*}E_{i}\left(t\right).$$

Here, *γ* is a damping rate (measured or computed by *ab initio* techniques), *ω*_0_ is the natural frequency, Z* is an effective charge called the mode-effective charge, and $E_{i}\left(t\right)$ is the time-dependent electric field (projected onto the direction of the dipole of the *i* phonon mode). The term $V_{NL}^{\prime }\left(\left\{Q\right\}\right)=\partial V_{NL}/\partial Q$ is a nonlinear force which can depend on other phonon modes. For example, in a down-conversion process, there could be a term in the potential *V*_*NL*_ of the form $Q_{i}Q_{j}^{2}$ which generates lower frequency phonons in mode *j*. Ignoring the nonlinear term for the time being, we may immediately see from Eq. (14) that in response to a time-harmonic drive of the (complex) form *E*_*i*_ = *E*_*i*,0_e^−i*ωt*^, the phonon mode will also undergo a coherent sinusoidal oscillation of complex amplitude

(15) $$Q_{0}=Z_{i}^{*}E_{i,0}/\left(\omega_{0}^{2}-\omega^{2}-i\omega \gamma \right),$$

which increases with the strength of the drive. As the drive strength increases, one can see from Eqs. (14) and (15) that the nonlinear terms become increasingly comparable to the linear terms, since they grow faster with the drive strength (for weak driving).

The electric fields needed to reach the nonlinear regime depend on material (which impacts the phonon dispersion and modes), as well as driving frequency and pulse duration. For infrared-active phonons in perovskite materials (e.g., LiNbO_3_, SrTiO_3_, KTaO_3_), the typical field scale needed to access nonlinearities with 100 fs pulses is on the order of 1 MV/cm. For longer pulses, the needed fields drop due to the mechanics of resonance, but also may lead to deleterious heating dynamics. A variety of nonlinear effects have been probed, arising from different terms in the nonlinear potential. The effects of these nonlinearities can be described in terms very familiar to those who have studied nonlinear optics. For example, a term of the form $Q_{1}^{2}Q_{2}$ can generate a second-harmonic mode *Q*_2_ (at frequency 2*ω*) when the material is driven by a field resonant with mode *Q*_1_ at frequency *ω*. Such effects have been demonstrated now in a variety of material platforms [48] and are promising as a form of creating controlled deformations of material lattices to induce control of functional properties of materials (electronic, optical, and magnetic properties, for example [49]). Although the distortions described thus far apply to every irradiated unit cell, being in essence uniform throughout the crystal, it is possible to engineer spatial variations in light-induced deformations, such as line-defects [50] and localized deformations that propagate through a structure [51].

**Figure 1:**
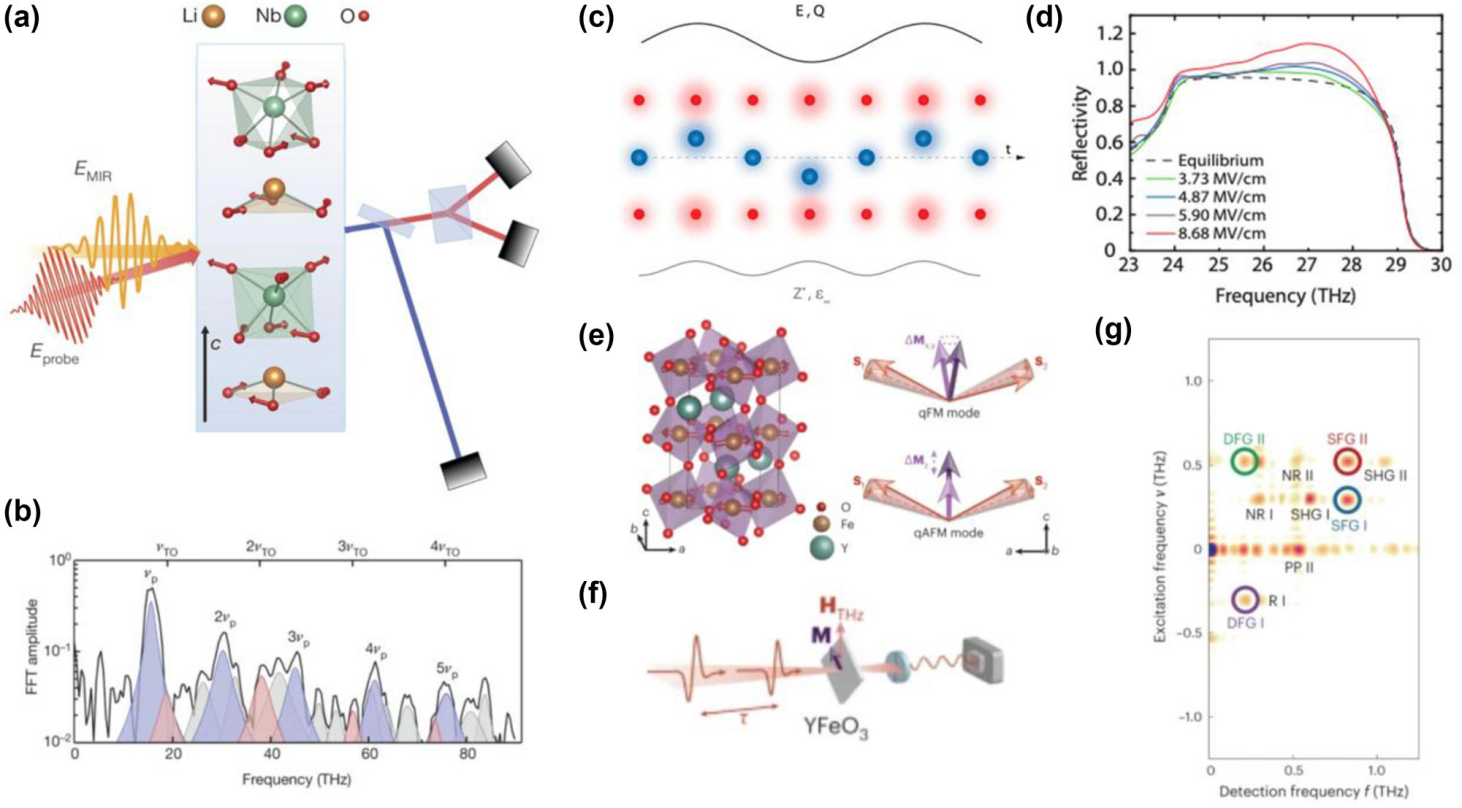
Terahertz nonlinearities in driven phonon and magnon systems. (a) Strong non-perturbative nonlinearities can be realized in polar insulators (such as lithium niobate, whose unit cell is pictured here), driven by intense terahertz pulses (the yellow MIR field denoted on the figure). The underlying material dynamics can be detected by a probe pulse (red). Nonlinear processes such as harmonic generation can be measured either by polarization rotation or second-harmonic generation of a probe pulse. (b) For strong driving fields, the driven phonon mode can oscillate at high harmonics of the driving field (here, as many as five). The black curves show the total spectrum. (c) In other polar systems, such as silicon carbide (whose lattice is shown by red and blue atoms), parametric down-conversion can be realized, converting a drive at frequency 2*ω* to a signal at *ω* (*E*, *Q*), which is manifested as amplification of a probe signal at frequency *ω*. Observing this amplification via reflection of the probe from the crystal entails measuring a reflectivity larger than one, which is seen in (d) for sufficiently strong driving fields that parametric down-conversion becomes efficient. The different curves represent the probe reflectivity for different driving fields. (e) Similarly to the case of phonons, strong terahertz drives can also excite nonlinear dynamics of magnons, as illustrated in the canted antiferromagnet YFeO_3_ (unit cell depected in (e)) which features quasi-ferromagnetic (q-FM) and quasi-antiferromagnetic (q-AFM) modes at different frequencies. The q-FM mode corresponds to precession of the net unit cell magnetization (purple arrow in upper right inset), while the q-AFM mode corresponds to a longitudinal modulation of the magnetization (lower right inset). (f) By driving this antiferromagnet with a magnetic field at an angle to the net magnetization, both modes can be excited, and if the drive is strong, they can nonlinearly mix, leading to sum and difference frequency generation (SHG, DFG) as well as second-harmonic generation (SHG) which can be observed via 2D terahertz spectroscopy as in (g). Figures (a, b) were adapted from Ref. [52] with permission from Springer Nature, while (c, d) were adapted from Ref. [53], and (e–g) were adapted from Ref. [54] with permission from Springer Nature.

Advances in generating strong fields at mid-infrared frequencies have made it possible to access even higher-order terms in the nonlinear potential of the lattice. For example, analogues of high-harmonic generation have been demonstrated, generating third-, fourth-, and even fifth-harmonics of optical phonons in lithium niobate [52], as illustrated in Figure 1a and b. Such higher-order polarization is readily detected optically.

The same nonlinear term ( $Q_{1}^{2}Q_{2}$ ) can also generate low-frequency phonons and even static rectification. For example, driving the mode *Q*_1_ with a pulse can lead to driving terms with a frequency equal to the difference between two frequency components of the original pulse (difference-frequency generation): such phonons generated can of course be at very low frequencies [55], [45], [56], [57]. It is similarly possible to generate zero-frequency components based on this approach, leading to a DC force on the mode *Q*_2_. It is possible for example to generate acoustic phonons through this coupling scheme. In another approach, if one drives the mode *Q*_2_ at a frequency defined as 2*ω*, it is possible to generate a mode *Q*_1_ at frequency *ω*. This process, which is the inverse of second-harmonic generation, is exactly analogous to parametric down-conversion in optics, and it (and analogues) has been observed in a variety of platforms including phonon polaritons in thin films of silicon carbide [53] cuprates [25], and excitonic insulator candidates [58]. In the latter case, the driven phonon couples to pairs of Josephson plasmons.

Based on the discussion in Section 2 on the relationship between squeezing and nonlinearity, one expects that these various forms of nonlinear dynamics should be capable of generating squeezing of different phonon modes. As a point of terminology, when we refer to squeezing of a particular phonon mode *i*, we mean that the variance of some particular quadrature goes below one in standard units (for the position quadrature, this means the position variance ${\left(\Delta Q_{i}\right)}^{2}<\hbar /2\omega_{i}$ , while for the momentum quadrature, this means ${\left(\Delta P_{i}\right)}^{2}<\hbar \omega_{i}/2$ . The dimensionless quadratures would then be defined as $Q_{i}/\sqrt{\hbar /2\omega_{i}}$ and $P_{i}/\sqrt{\hbar \omega_{i}/2}$ ). Previously, the term squeezing has also been used interchangeably with having a variance that oscillates at twice the frequency of the phonon mode. Although such a 2*ω* oscillation is required by squeezing (as discussed in Section 2), it does not necessarily entail a variance which goes below unity.

**Figure 2:**
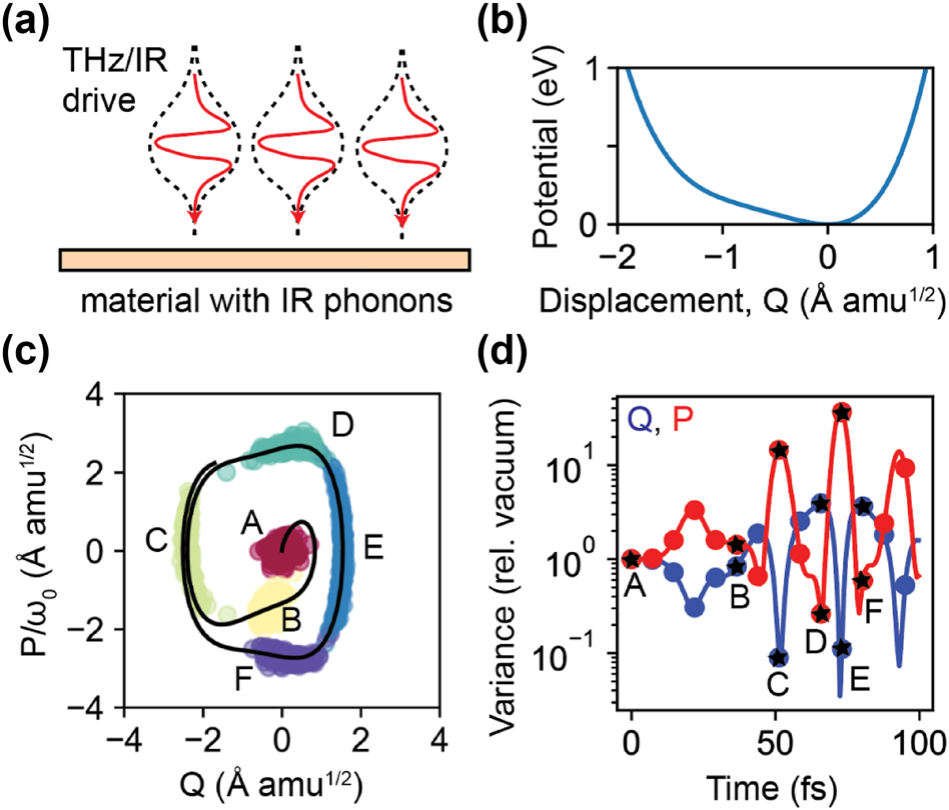
Squeezing and correlations in nonlinear dynamics of light-driven optical phonons. (a) A few-cycle terahertz pulse incident in a material can resonantly excite a phonon mode, causing displacement of the atoms in the lattice. The material considered in this example is LiNbO_3_. (b) Potential energy as a function of modal displacement *Q*. The analytical form of the potential is given in Eq. (16) below. For large displacements, the potential deviates strongly from the harmonic approximation typically used to describe phonon dynamics. (c) Dynamics of the modal displacement and the conjugate modal momentum (*P* = d*Q*/d*t*), with *ω*_0_ being the phonon frequency in the harmonic approximation. Black line indicates mean trajectory. Red dots represent a cluster of initial conditions, and dots of different colors represent how those initial conditions evolve over time (letters have the same meaning in (c) and (d)), showing the spread of (quantum) fluctuations in the initial conditions. (d) Variance of modal displacement (blue) and momentum (red), calculated using quantum sensitivity analysis, showing large degrees of squeezing of the modal displacement (above 10 dB), resulting from a strong insensitivity of the displacement to the initial conditions for certain times. The driving pulse is taken to be $E\left(t\right)=E_{0}\sin\left(\omega t\right){\text{e}}^{-t^{2}/\tau^{2}}$ , with *E*_0_ = 100 MV/cm, *ω*_*d*_ = 2*π* × 17.5 THz, and *τ* = 150 fs. Data generated for this figure is simulated using the quantum sensitivity analysis framework discussed in Section 2 and derived in the Supplementary Information. Code which generates and plots the data is provided in: https://github.com/nrivera494/Phonon-squeezing-by-ultrafast-driving.

Borrowing from the deep knowledge of quantum light generation that has been accumulated in optics, the most obvious candidate to realizing squeezing in phononic platforms is in non-centrosymmetric materials (e.g., materials in their ferroelectric phase), where the nonlinear potential can contain cubic terms of the form described above. However, squeezing effects are not generic, requiring a phonon band which is at half the frequency of the driven band. Unlike in photonics, where phase matching can be engineered by material geometry, the phononic dispersion properties are much less straightforward to tune. While by no means prohibitive to achieving squeezing, it is important to find more generic approaches to squeezing that can apply in principle to any material. For example, approaches that require only a single phonon band, as we will consider in Figure 2.

In Figure 2, we argue that squeezing can be realized generically in driven phononic systems that have a strong enough driving field to realize nonlinear effects, within the decay time of the phonons – even with a single band – where second-order nonlinearity is not particularly important. As an example, we take the system considered in Figure 1a and b: we consider a material (here, LiNbO_3_) in the presence of a strong mid-IR drive which excites an optical phonon (of frequency 15 THz). The optical phonon corresponds to a displacement of the *κ* atom of each unit cell, which is typically parameterized by *Qη*_*κ*_, where *η*_*κ*_ is an atom-dependent vector of eigendisplacements. The modal displacement *Q* is defined as previously discussed, such that potential energy is given by

(16) $$V\left(Q\right)=\frac{1}{2}\omega_{0}^{2}Q^{2}+\frac{1}{3}a_{3}Q^{3}+\frac{1}{4}a_{4}Q^{4}+\frac{1}{5}a_{5}Q^{5}$$

(all parameters are taken from Ref. [52]). We note that while there is a cubic term here, corresponding to second-order nonlinearity, the effect we will show persists at a similar magnitude even when the cubic term is removed.

The classical trajectories associated with various initial conditions are shown in Figure 2c, and show the existence of certain times for which the variance of *Q* or *P* can be well below the standard quantum limit. Examining the mean trajectory (in black), it is clear why this can happen. The mean trajectory has a quasi-rectangular shape. In the relatively flat regions, either *Q* or *P* hardly changes over time. For example, around the region in phase space near time *C*, *Q* hardly changes with time. Given that a time-delay (or phase lag) is similar to a change in initial conditions, this indicates that different initial conditions lead to minimal changes in *Q*, leading to lower noise than the standard vacuum level (as per quantum sensitivity analysis). This is shown in Figure 2d where using quantum sensitivity analysis, we find certain times for which the lattice fluctuations can be suppressed by over 10 times the vacuum level. There are also times for which fluctuations in the modal momentum can be very large, and as large as the mean values themselves, indicating the buildup of macroscopic fluctuations.

These results are interesting in light of the fact that substantial phonon squeezing has yet to be measured, but may already be present in existing experiments. This would also give a route to generate squeezed light at mid-IR and THz frequencies, where squeezed sources have not been developed. THz light generation would be achieved by exploiting the coupling of these IR-active phonons to light, manifesting as phonon polaritons which could be outcoupled to the far-field. The same far-field coupling could enable routes to detect quantum noise dynamics of phonons as well. By being able to map the quantum statistics of the phonon fields to that of the radiated light, one can use the measured light to infer the underlying quantum dynamics of the material: this program is sometimes referred to as noise spectroscopy, and is conventionally a powerful tool to understand dynamics which are not apparent from mean-field measurements [59].

The detection of quantum fluctuations of the electromagnetic field is typically done via techniques such as homodyne detection, for which there has been much work done at optical and infrared frequencies. At the same time, special techniques have been developed for detecting quantum noise in terahertz (and mid-infrared) fields, which exploit electro-optic sampling instead of balanced detection via photodiodes [43], [60], [61], [62], [63]. These results are also interesting given that generally, high-harmonic phonon generation as a route to squeezing has not been looked at, as it is typically assumed that one would need a parametric interaction or a Kerr interaction to squeeze. We emphasize that the perspective from quantum sensitivity analysis not only makes clear the physics in a way that a standard approach does not, but it also indicates a clear classical guideline to reducing the fluctuations further. For example, by engineering the driving field to induce a more rectangular trajectory, fluctuations could be suppressed further. This highlights an important role for drive-engineering as well in generating quantum states of material excitations at new frequencies.

We note that besides using these phononic squeezing dynamics to generate quantum light at terahertz frequencies, phonon squeezing may also be used as a tool to control material properties such as magnetism, optical properties, and even superconductivity. Intuitively, the importance of phonon squeezing for these properties comes from the fact that material properties are strongly shaped by electron-phonon and spin-phonon coupling. Even in the absence of real phonons (e.g., at low temperatures, which for optical phonons, often coincides with room temperature), vacuum fluctuations are relevant. For example, virtual phonon emission and re-absorption mediate electron-electron interactions as in superconductivity, as well as control the band-gaps of semiconductors and insulators. In the case of superconductivity, it has been argued theoretically that phonon squeezing (multimode two-mode squeezing of Raman phonons, in particular) can enhance the superconducting transition temperature, possibly explaining measurements of superconducting signatures in the optical properties of driven materials [23]. Although Raman phonons are not readily directly excited by light, they can be excited by an optical phonon, the latter of which can be coherently excited by light. I briefly also mention another tantalizing possibility: engineering these vacuum fluctuations by means of optical cavities, and controlling both phononic fluctuations and material phases such as ferroelectrics, as recently theoretically proposed in Ref. [64].

To conclude the discussion of phonons, we briefly mention that optical phonon driving is not the only way to achieve squeezing of phonons. Another class of techniques are “impulsive” approaches where an ultrashort but very intense pulse instantaneously changes the electronic properties of the system, manifesting as an abrupt change in the phonon frequencies [30], [31]. In general, such approaches have not unambiguously demonstrated phonon variances much below the shot noise level, but should be capable of doing so. That said, the impulsive stimulated Raman scattering effect upon which this squeezing is based is generally inefficient, and the short duration of driving also tends to inhibit squeezing, which may make approaches based on coherent driving of optical phonons ideal.

Conceptually similar progress to the case of phonons is now also being realized for magnons, which can also be driven resonantly by terahertz pulses. Magnons, or spin waves, are collective excitations corresponding to wavelike disturbances where the local magnetization is modulated. This is in analogy to phonons, where one has a wavelike disturbance of the positions of the atoms which make up the lattice. Magnons, being spin waves, respond efficiently to the magnetic field of a terahertz pulse, which can drive either longitudinal oscillations of the local magnetization or transverse oscillations (leading to precession of the magnetization), as shown in Figure 1e and f. For sufficiently strong driving fields, these magnon modes can mix due to nonlinear terms which are higher-than-quadratic order in the magnetization. This can lead to upconversion, as well as sum- and difference-frequency generation, all of which have been recently observed in antiferromagnets [54], [65]. Similarly to the case of phonons, the nonlinearity is described by nonlinear classical equations of motions for interacting magnons. One expects that the same type of interactions that enable sum and difference frequency generation should allow for parametric down-conversion and also self-phase modulation, each of which would allow for squeezing and entanglement of magnons. Similarly to the case of optical phonons sketched above, the quantum sensitivity analysis framework should elucidate which magnon observables are squeezed, and should also elucidate which type of nonlinear terms will be most effective in generating quantum states of magnons.

Generating quantum states of magnons opens up a wide variety of new possibilities. For example, squeezing of magnons could lead to squeezed light in the frequency range of 1 THz and even below. These frequencies can also interface with electronics. Further, quantum states of magnons may also have implications for spin-electron and spin-phonon coupling, allowing the enhancement or suppression of various relaxation and dephasing processes mediated by coupling to spin. Further, squeezing of spin fluctuations could lead to stabilizing or destabilizing various magnetic orders relative to the case of thermal equilibrium.

## 4 Quantum optics at X-ray frequencies using high-harmonic generation

In this section, we discuss another important class of nonlinear effects, called high-harmonic generation, that converts part of a strong infrared pulse into ultraviolet and even X-ray photons. This process, which was discovered in the 1990s, and recently acknowledged by the 2023 Nobel Prize in Physics, has been realized by many groups worldwide, in gases, solids, and even liquids. The process is famous for realizing attosecond pulses of light [66]. The attosecond nature of the generated light can be understood simply as follows: high-harmonic generation can be seen as a three-step process [67]. In the first step, a strong electric field strongly tilts the Coulombic potential binding an electron to an atomic nucleus, resulting in an electron in the ground state being able to tunnel out of the atom. In the second, the newly-liberated electron undergoes driven motion due to the strong oscillating driving field, which is well understood classically in terms of a free electron in an AC electric field, accelerating and gaining significant energy. In the third step, the electron can recombine with the nucleus, emitting a high energy photon in the process. This three-step process occurs with the periodicity of the drive, and so the emitted light has spectral content at integer multiples of the drive frequency. Because of the symmetry of the atomic potential, only odd harmonics are typically produced (with exceptions) which are coherent with each other. The resulting waveform is them a frequency comb whose pulse duration is dictated by the overall span of the comb. The span of the comb is determined by the atomic potential and the driving field, but for sufficiently strong fields, can span hundreds of harmonics of the driving field, leading to UV and even soft X-ray photons being generated.

For the first 20 years of research in high-harmonic generation, a semi-classical picture of the process has sufficed. In particular, many phenomena in HHG can be well-understood by quantizing the electron but treating the electromagnetic field classically [68]. In this picture, the radiation is understood as governed by the classical Maxwell equations with a source. The source is simply the time-dependent expectation value of the dipole moment of the radiating atoms (or for solid-state electrons, the time-dependent expectation value of the current density). This time-dependent dipole or current is found by solving the time-dependent Schrodinger equation in the presence of a classical driving field either based on simple model potentials or from density functional theory [69], [70].

**Figure 3:**
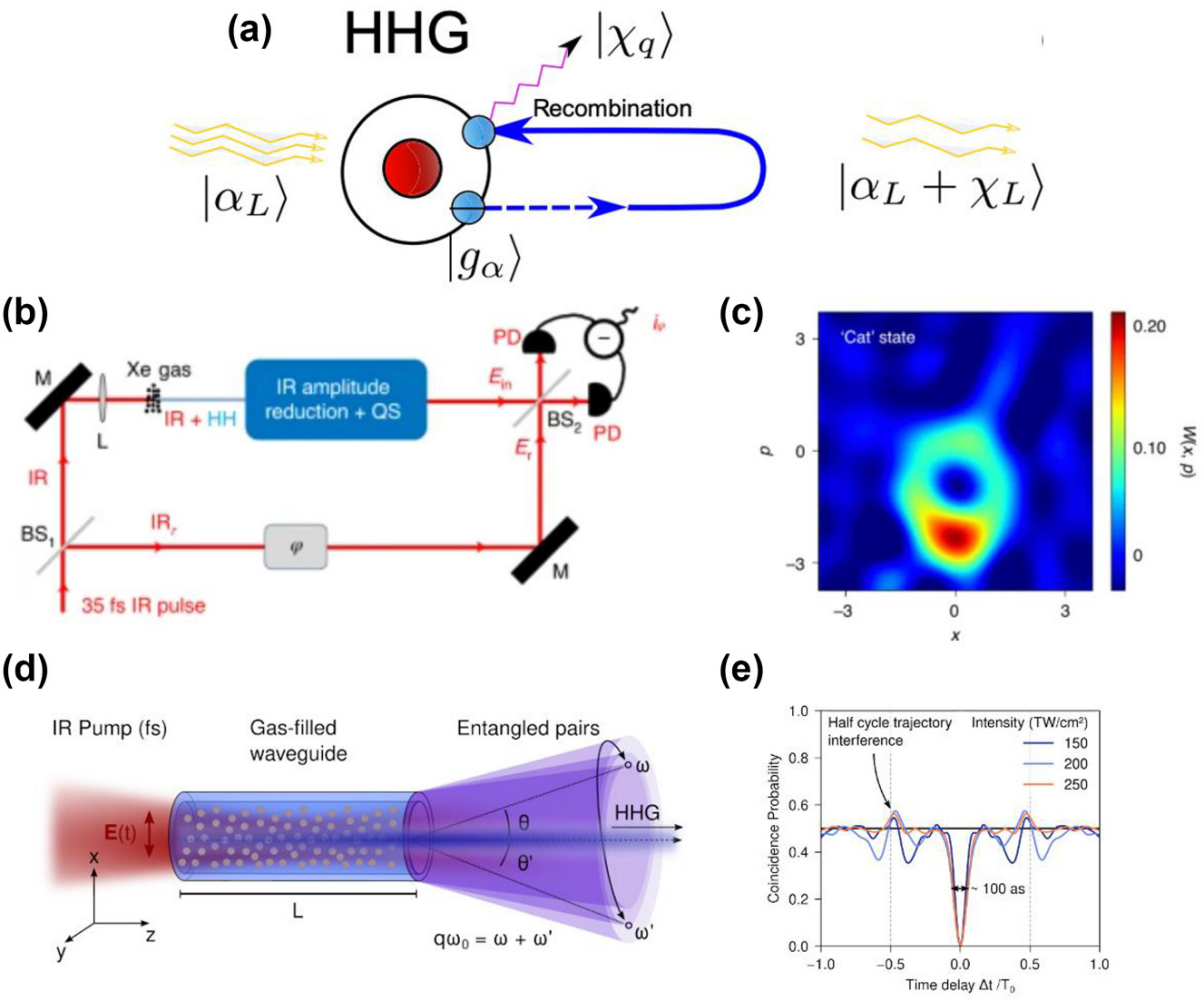
Quantum optics in high-harmonic generation. (a) The quantum-optical description of “conventional” high-harmonic generation: a driving laser field, which is thought of as being in a quantum-mechanical coherent state $\left.\left|\alpha_{L}\right.\right\rangle$ , drives an atom initially in its ground state, initiating a three-step process of electron (blue circle) tunneling, field-induced acceleration, and recombination with a parent ion (red). In the quantum description of high-harmonic generation, the driving field and the emitted harmonics $\left.\left|\chi_{q}\right.\right\rangle$ are all in coherent states, while the driving field under goes a displacement to a state $\left.\left|\alpha_{L}+\chi_{L}\right.\right\rangle$ . (b) Method of generating quantum light by post-selection. By filtering and measuring the output of HHG (top arm of the interferometer), it is possible to produce a non-classical superposition of coherent states. Its Wigner function can be measured by homodyning the filtered output of HHG with a local oscillator, and a typical Wigner function of a superposition of coherent states is shown in (c), $W\left(x,p\right)$ , where *x*, *p* are position quadratures of the light field at the drive frequency. The negative values are a signature of non-classicality. (d) A proposed method for generating entangled X-rays from high-harmonic generation based on a two-photon process in which the recombining electron in (a) emits two photons instead of one (a second-order process in quantum electrodynamics). The figure shows a gas pumped by a strong IR drive in a waveguide geometry to facilitate phase-matching of the two-photon process. The two photons have quantum correlations which can be probed by a Hong–Ou–Mandel experiment. A prediction of coincidence probability for the photon pairs is shown in (e), which shows perfect destructive interference of coincidences for pairs less than 100 as apart in time, for different intensities of the drive (corresponding to different colored lines). Figure (a) is adapted from Ref. [73], while (b, c) are adapted from Ref. [72] with permission from Springer Nature and (d, e) are adapted from Ref. [74].

Recently, there has been interest among several groups worldwide in quantizing the electromagnetic field, and understanding the signatures of quantum optics in high-harmonic generation. Some of the earlier work along these lines was focused on reconciling the accuracy of the classical description of HHG with the fundamental description according to a fully quantized theory. The consensus is that for a driving field which is in a coherent state, the induced current experiences only weak fluctuations, closely approximating a classical current, which generates coherent states of the different harmonics – with zero quantum correlation between the harmonics. In other words, the output state of the light is a product state of coherent states for the different harmonics, with the coherent state amplitudes governed by the classical Maxwell equations [71], [72]. This is illustrated schematically in Figure 3a.

Although, from this perspective, HHG seems classical, there are a number of routes to generating genuinely quantum light through HHG. Generating quantum light at X-ray frequencies, while also of clear fundamental importance, may also lead to applications in fields like X-ray imaging (where shot noise is a limitation) [75] and X-ray spectroscopy of solids and biomolecules, where the often high X-ray fluxes needed to get signal lead to sample damage [76].

Many approaches to generating quantum light from HHG follow a close analogy with methods for generating quantum light at optical frequencies. For example, even in the case where coherent states are generated, measurements on the output state of the harmonics can herald a non-classical superposition of coherent states (of which the Schrodinger cat state is an example) [72], [73], [77]. A proof-of-concept demonstration of this effect was put forth early in this field (Figure 3b and c).

Beyond this, most other approaches remain only theoretically predicted for now. These newly predicted effects, for the most part, have the major advantage that the quantum light is produced deterministically. For example, by exploiting high-harmonic generation in the depleted regime, where a significant portion of the incident drive gets converted into harmonics, the fundamental harmonic can be squeezed [78]. This is in analogy to the case in optics, where in second-harmonic generation, the fundamental harmonic can develop squeezing [79]. In another example, which is conceptually related, it has been predicted that for sufficiently strong driving fields, the recombining electron emitting HHG photons can emit pairs of photons with quantum correlations [74] (Figure 3d and e). Such two-photon HHG can be seen as a low-gain version of a parametric amplifier which generates squeezing in optics. Here, the major difference in this proposed scheme, from the depleted-regime case described above, is that even the X-ray harmonics can have strong quantum correlations. Beyond these examples, many others have emerged even in the last year, including examples of using inter-atomic correlations to generate quantum HHG light [80], [81].

**Figure 4:**
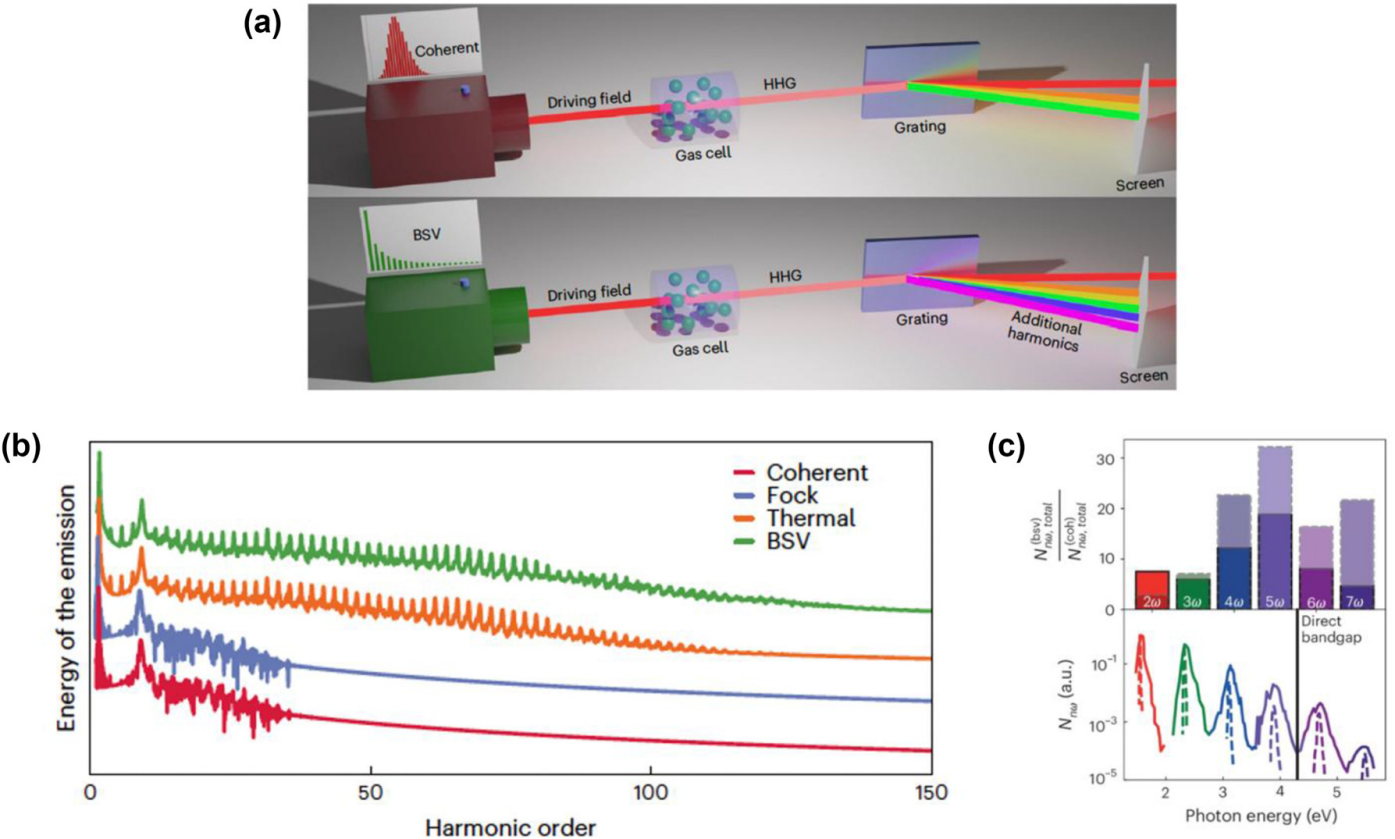
Driving high-harmonic generation (HHG) with quantum light. (a) Typically, HHG is driven by light which can be thought of as being in a quantum-mechanical coherent state. However, one can consider driving HHG with intense light with different statistics, such as bright squeezed vacuum or even thermal statistics. (b) When looking at the spectrum of HHG generated by different quantum states (coherent, Fock, thermal, and BSV) with the same mean intensity, BSV and thermal light can generate much higher harmonics, beyond the conventional cutoff associated with coherent light. This is because thermal and BSV light have strong intensity fluctuations, and so their intensity can in principle be much higher than the mean intensity. (c) Experimental proof-of-concept for the effect, suggesting that harmonic generation in solids can be more efficient when driven with BSV-like light, compared to coherent light of the same intensity. (a, b) Is adapted from Ref. [82], while (c) is adapted from Ref. [84]. (a–c) are modified with permission from Springer Nature.

Before concluding this section, we mention a different flavor of quantum HHG which segues into the next section. The examples above were focused on driving the HHG source (be it a gas or a solid) with classical light (coherent states), and exploring the quantum nature of the emitted light. We can also ask about *driving* HHG with quantum light, and more broadly, the role of the statistics of the driving light on the emitted light. One of the first studies on this effect found that by driving HHG with light that has macroscopic intensity fluctuations (such that the uncertainty in the intensity is similar in magnitude to the mean intensity), the spectrum of the emitted HHG can be strongly extended, supporting a much larger number of harmonics compared to the case when driving HHG by a coherent state of the same mean intensity [82] (Figure 4). Examples of light with macroscopic intensity fluctuations include thermal light, as well as bright squeezed vacuum (the squeezed vacuum state described in Section 2, but with a mean number of photons much larger than 1. In other words, with *r* very large.). This effect is not in-and-of-itself quantum, and relies mostly on the fact that the output HHG spectral intensity rises rapidly with the intensity of the driving light. In that case, the effect of intensity fluctuations away from the mean gets enhanced. Since in the case of both thermal light and bright squeezed vacuum, there is a significant probability of having an intensity twice or even thrice the mean value, it is as if the HHG is driven by an effectively higher intensity, which in the classical case, leads to higher frequency output. Such effects have some precedent in perturbative nonlinear effects such as second-, third-, and fourth-harmonic generation seeded by bright squeezed vacuum [83], and proof-of-concept experiments have emerged for these effects [84]. Further explorations into the role of multimode correlations are presently underway [85].

It is important to emphasize that this effect can be understood in a semi-classical way, as the same effect can be realized by driving with intense thermal light, which is effectively classical insofar as a thermal state can be represented as an incoherent mixture (probability distribution) of coherent states [86]. The same effect can also be realized by driving the HHG with an intense laser with extremely high intensity fluctuations, and the origin of the effect is ultimately the strong sensitivity of the high-harmonics fluctuations in the intensity of the driving laser. I further emphasize that definitive quantum signatures, such as quantum coherence, at the output of HHG, at the time of this writing, remain to be explored. Indeed, the broader question of how the *quantum state* (rather than the mean intensity) of the output light depends on the quantum state of the driving field, remains relatively wide open for exploration.

We conclude this section by describing some of the likely and important future directions of exploration in the quest for quantum light at ultraviolet and X-ray frequencies. One thing that is missing from the discussion above is strongly squeezed light at the harmonic frequencies. Squeezing approaches based on depletion or post-selection described above apply to the lower harmonics, or the fundamental. The two-photon HHG approach theoretically predicted is analogous a low-gain parametric amplifier: the squeezed state described earlier, of the form $\left|\psi \right\rangle ∼{\text{e}}^{ra^{†2}}\left|0\right\rangle$ is, for *r* ≪ 1, a superposition of a vacuum state and the two-photon state (with a probability proportional to *r* ^2^). The probability of generating two-photons in the two-photon HHG process described in Ref. [74] is much smaller than one, and is analogous to the weakly-squeezed state described above. Generating something closer to the squeezed states used in metrology at optical frequencies requires making the two-photon nonlinearity more efficient, through the use of feedback cavities (e.g., based on highly efficient Bragg mirrors at X-ray wavelengths) or solids, which generate HHG more efficiently than in gases.

Another important question is related to the effect of noise in the driving lasers which induce HHG. Lasers at these ultra-high intensities, as will be mentioned in the next section, tend to have noise levels far in excess of the level associated with coherent states (often by orders of magnitude). At sufficiently high noise levels of the drive, the output harmonics of the HHG process will not exhibit squeezing. There is therefore an important question as to how the noise in the drive, as well as quantum noise in the harmonics, affects the overall quantum state of HHG. Finally, we should mention that beyond HHG, there are other effects capable of generating X-rays with quantum properties. For example, free-electron lasers produce intense X-ray beams which can undergo nonlinear dynamics in materials. Exploiting those nonlinear dynamics could provide a route to generating squeezed X-rays as well. In all of these cases, the quantum sensitivity analysis framework described in this work should allow insight into all of these questions and more.

## 5 Outlook

The development of new platforms for nonlinearity, at terahertz frequencies and even X-ray frequencies, as I’ve argued above, gives promising new avenues for generating quantum states of light at new frequencies, as well as quantum states of material quasiparticles. What all of these nonlinear platforms have in common is that they require driving by fairly intense lasers. I’ve also shown that for the case of phonons, even fairly low quality factors (approximately 10) do not prohibit the squeezing from happening. We expect that to also be the case for magnons. Nevertheless, there are a number of important questions that will need to be resolved before such effects can be realized and exploited in earnest. *In fact, as we’ll argue now, this field will require developments in fundamental laser physics and technology.*

In the standard quantum optical description of strongly-driven systems, it is almost universally assumed that the driving field is in a coherent state. This assumption is nearly always violated in practice with high-power lasers. A simple way to see this is that many high-power lasers are realized by amplifying a lower-power laser (e.g., via an erbium-doped amplifier or a rare-earth-doped laser crystal). Assuming that the lower power pulse is in a coherent state, the amplified light will have intensity fluctuations in the total photon number

(17) $${\left(\Delta n\right)}^{2}=\left(2G-1\right)\left\langle n\right\rangle ,$$

where $\left\langle n\right\rangle$ is the number of photons at the output of the amplifier, and *G* is the power gain. The Fano factor $F\equiv {\left(\Delta n\right)}^{2}/\left\langle n\right\rangle =2G-1$ , in contrast to a coherent state, where this ratio is 1 (corresponding to Poissonian statistics). As is seen from Eq. (17), for large gains, the intensity (and also phase) fluctuations of the amplified light are large, and this noise can transfer to the system that this laser is used to drive, be it phonons or magnons or a gas undergoing high-harmonic generation. For large gains typical of millijoule and several microjoule pulses, this noise transduction can be substantial.

The discussion of the previous paragraph raises a larger question as to what noise characteristics are required for the driving fields in order to realize states that are genuinely quantum. In fact, this is currently a frontier area in laser physics, with many theoretical and experimental questions. For example, there are few measurements of quantum noise of very high-power lasers. In part, that is because of a paucity of low-noise detection systems that can handle high powers. Typically, quantum statistics of light in the infrared and visible regime would be determined by photocurrent statistics in different configurations (direct detection, balanced intensity detection, homodyne, etc.). The photocurrent is typically generated by illuminating a photodiode. But most photodiodes saturate at average powers which are quite modest compared to those used to drive the material systems discussed in this work.

Another major question is related to the limits of noise suppression. Given a system which is far from being in a coherent state (i.e., for an amplified laser), is it possible to suppress noise to, or even below the shot noise level associated with coherent states? Recently, this question has been broached, where it has been shown that for femtosecond pulses with noise well-above the shot-noise level (10 dB or 10× more noise than a coherent state), nonlinear filtering can strongly reduce the noise, generating squeezed light with noise 4 dB below that of a coherent state: the total attenuation was roughly four times less than needed from linear attenuation to get the same variance (linear attenuation reduces the intensity noise as well as the average intensity) [40], [41]. This is due to an “attractor” effect, in which the output of the nonlinear filtering process is strongly insensitive to changes in the initial pulse incident into the nonlinear filter.

In general, an answer to the question of how to produce high-power lasers which are close enough to being coherent states is wide open, since it requires understanding quantum noise in systems that are often nonlinear (due to large powers), multimode (spatially and temporally), and non-conservative (due to gain and loss). Along these lines, there have been a number of developments in numerical tools and theoretical techniques to understand noise dynamics in nonlinear and multimode systems [87]. Beyond that, a number of new platforms have been explored in the context of understanding the fundamental physics of quantum noise, from soliton microcombs [9], [88], [89], to multimode fibers [90], supercontinuum generation [40], and pulsed optical parametric oscillators [91]. On the theory side, a number of suggestions have recently been proposed for creating high-power lasers with noise at or below the quantum shot-noise limit. Approaches include solid-state [92] and semiconductor gain media in cavities [93] with Kerr nonlinear media (leading to nonlinear gain which adds less noise than conventional linear gain), as well as nonlinear cavities with a frequency-dependent outcoupler that can do not outcouple for certain intracavity intensities (representing a type of nonlinear loss) [94], [95]. This latter approach can be thought of as a passive feedback scheme that autonomously keeps the intensity at a certain well-defined level.

Once these questions are resolved, several exciting opportunities should open up based on the generation of squeezed and low-noise systems at terahertz and X-ray frequencies. For example, squeezing at terahertz frequencies could open up opportunities in molecular spectroscopy and terahertz communications, while squeezing at X-ray frequencies could open up opportunities in X-ray imaging (where shot noise is a limitation) [75] and X-ray spectroscopy of solids and biomolecules. In this latter case, squeezing could be of interest if extended to suitably high X-ray fluxes, as squeezing would lower the powers needed to do spectroscopy, which is important since many systems and materials get damaged by X-ray fluxes needed for spectroscopy measurements [76].

## Supplementary Material

Supplementary Material Details
