# Supplementary material for: New opportunities for creating quantum states of light and matter with intense laser fields
Source: Nanophotonics. 2025 Mar 31;14(11):1837–55. doi: 10.1515/nanoph-2024-0605 (PMC12133219; doi:10.1515/nanoph-2024-0605)
Supplement: Supplementary file 1 — Supplementary Material Details [file j_nanoph-2024-0605_suppl_001.pdf]

Supplementary Information for:

New opportunities for creating quantum states of light and matter with intense laser fields

Nicholas Rivera[1,2*]

[1] *Department of Physics, Harvard University, Cambridge, MA 02138, USA.*

[2] *School of Applied and Engineering Physics,*

Cornell University, Ithaca, NY 14853, USA. *

*Electronic address: nrivera@fas.harvard.edu.

Contents

S1. The quantum optical law of total covariance and quantum sensitivity analysis 2

S1.1. Application to shot-noise limited inputs 5

S1.2. Case of an input-output theory specified by position and momenta 6

S2. Applying the framework to textbook problems in quantum optics 7

S2.1. Quantum noise in linear dissipation 7

S2.2. Continuous dissipation 8

S2.3. Parametric amplification 10

S2.4. Linear amplifier 11

S2.5. Kerr squeezing 12

References 13

S1. THE QUANTUM OPTICAL LAW OF TOTAL COVARIANCE AND QUANTUM SENSITIVITY ANALYSIS

In this note, we derive a general formulation of quantum noise problems in nonlinear optics, in the regime where the incident light has many photons in it. The derivation we present below applies to more than interacting systems of light: as will be seen, our derivations also apply to any systems of bosons, and thus also cover the case of light interacting with matter degrees of freedom. The net result of this new formulation is what we call the quantum optical law of total variance. It allows us to express *any* quantum noise – in a strongly-interacting, multimode system, consisting of light and matter degrees of freedom – in terms of a sum of independent variances coming from the effect of vacuum fluctuations in each mode of quantized light and matter fields.

Consider a system of bosons described by a set of annihilation and creation operators $a_i, a_i^\dagger$ where i is a generalized index, labeling not only different modes for a given boson, but also different types of bosons. We'll denote the vector of operators as $\mathbf{a}, \mathbf{a}^\dagger$ for compactness. For example, in a case where light interacts with phonons, as well as atoms capable of absorbing the light, the boson operators may label photon modes, phonon modes, and the effective bosonic modes which describe an absorbing material.

The equation of motion for the operators can be written schematically as:

$$\dot{\mathbf{a}} = F(\mathbf{a}, \mathbf{a}^\dagger)$$
$$\dot{\mathbf{a}}^\dagger = F^\dagger(\mathbf{a}, \mathbf{a}^\dagger), \tag{S1}$$

where F is a generic operator function that produces the right-hand side of the Heisenberg equations of motion corresponding to the system Hamiltonian.

In the case where the number of bosons in the initial state is large, the quantum dynamics can be well approximated by the lowest-order fluctuations on top of the mean-field dynamics. This "linearization" approximation proceeds by expressing the operators as

$$\mathbf{a} = \boldsymbol{\alpha} + \delta\mathbf{a}, \tag{S2}$$

where $\boldsymbol{\alpha} \equiv \langle \mathbf{a} \rangle$, plugging it into the Heisenberg equation, and neglecting terms of higher order than linear in $\delta\mathbf{a}$. The equation of motion for the mean fields can be written as

$$\dot{\boldsymbol{\alpha}} = F(\boldsymbol{\alpha}, \boldsymbol{\alpha}^*), \tag{S3}$$

where the c-number function $F(\boldsymbol{\alpha}, \boldsymbol{\alpha}^*)$ corresponds to replacing all operators in $F(\mathbf{a}, \mathbf{a}^\dagger)$ by c-numbers. These equations will be nothing other than the classical equations of motion for the system. Meanwhile, the equation of motion for the fluctuations can be expressed as

$$\delta\dot{a}_i = \sum_j \frac{\partial F_i}{\partial \alpha_j} \delta a_j + \sum_j \frac{\partial F_i}{\partial \alpha_j^*} \delta a_j^\dagger \tag{S4}$$

where, importantly, the derivatives are with respect to the classical (c-number) function. The equation of motion for the creation operators just follows from conjugation.

The solution to these equations can be expressed as a Bogoliubov transformation, as:

$$\delta a_i(t) = \sum_j \mu_{ij} \delta a_j(0) + \nu_{ij} \delta a_j^\dagger(0). \tag{S5}$$

In what follows, we will make a new connection, showing that the μ, ν are in fact derivatives of the classical equations of motion with respect to the initial conditions. To see this, let us consider when we take the classical equation of motion Eq. S3 and evaluate the change in the solution when the initial conditions are varied. Assuming that a variation in the initial conditions, $\delta\boldsymbol{\alpha}$ leads to a sufficiently small change in the output fields, we may linearize the equations, writing them as

$$\delta\dot{\alpha}_i = \sum_j \frac{\partial F_i}{\partial \alpha_j} \delta\alpha_j + \sum_j \frac{\partial F_i}{\partial \alpha_j^*} \delta\alpha_j^* \tag{S6}$$

This equation is in correspondence with Eq. S4, and its solution is the same, as Eq. S4 is a linear equation, and thus is solved identically to a classical equation. In particular, we may write:

$$\delta\alpha_i(t) = \sum_j \mu_{ij}\delta\alpha_j(0) + \nu_{ij}\delta\alpha_j^\dagger(0), \tag{S7}$$

where μ, ν are identical to the quantum-mechanical case. But, by construction, we may also write

$$\delta\alpha_i(t) = \sum_j \frac{\partial\alpha_i(t)}{\partial\alpha_j(0)}\delta\alpha_j(0) + \frac{\partial\alpha_i(t)}{\partial\alpha_j^*(0)}\delta\alpha_j^*(0), \tag{S8}$$

allowing us to identify

$$\mu_{ij} = \frac{\partial\alpha_i(t)}{\partial\alpha_j(0)}, \nu_{ij} = \frac{\partial\alpha_i(t)}{\partial\alpha_j^*(0)}, \tag{S9}$$

completing the proof.

We now use this result to connect the calculation of quantum noise in multimode systems of light and matter to adjoint methods for solving numerical differential equations. Consider an observable

$$X = \sum_i c_i a_i + d_i a_i^\dagger. \tag{S10}$$

In terms of the results of the previous section, we may write

$$\begin{aligned}
\delta X(t) &= \sum_{ij} \left[c_i \frac{\partial\alpha_i(t)}{\partial\alpha_j(0)} + d_i \frac{\partial\alpha_i^*(t)}{\partial\alpha_j(0)}\right]\delta a_j(0) + \left[c_i \frac{\partial\alpha_i(t)}{\partial\alpha_j^*(0)} + d_i \frac{\partial\alpha_i^*(t)}{\partial\alpha_j^*(0)}\right]\delta a_j^\dagger(0) \\
&= \sum_j \frac{\partial X(t)}{\partial\alpha_j(0)}\delta a_j(0) + \frac{\partial X(t)}{\partial\alpha_j^*(0)}\delta a_j^\dagger(0) \\
&\equiv \left[\delta\boldsymbol{a} \cdot \frac{\partial}{\partial\boldsymbol{\alpha}} + \delta\boldsymbol{a}^\dagger \cdot \frac{\partial}{\partial\boldsymbol{\alpha}^*}\right] X(\boldsymbol{\alpha}, \boldsymbol{\alpha}^*)
\end{aligned} \tag{S11}$$

In the last line, and in what follows throughout this Supplement, we will omit explicit indication of time-zero quantities. We also briefly note that in the main text, for intuitive ease, we have replaced the time-zero label by "in" and the time-t label by "out". Further, in the last line, we have introduced the notation $X(\boldsymbol{\alpha}, \boldsymbol{\alpha}^*)$, which refers to the value of the observable X, calculated *classically*, assuming some initial conditions $\boldsymbol{\alpha}, \boldsymbol{\alpha}^*$. While classically, the initial conditions and their conjugates would not be independent, here, we take them as independent, since they refer to fluctuations in a and $a^\dagger$, which quantum-mechanically have a nonvanishing commutator.

Since this expression applies to any observable, we may now write a compact relation for the variance in an arbitrary observable, in an arbitrary coupled system of light and matter degrees of freedom, in this linearization approximation. This relation is what we refer to as the quantum

optical law of total variance. Within this approximation, the variance of X, $(\Delta X)^2 = \langle(\delta X)^2\rangle$, is expressed as a quadratic form:

$$\begin{aligned}(\Delta X)^2 &= v^T C v, \text{where} \\ v &= \begin{pmatrix} \frac{\partial X}{\partial \boldsymbol{\alpha}} & \frac{\partial X}{\partial \boldsymbol{\alpha}^*} \end{pmatrix}^T \\ C &= \begin{pmatrix} \langle \delta\mathbf{a}\delta\mathbf{a}\rangle & \langle \delta\mathbf{a}\delta\mathbf{a}^\dagger\rangle \\ \langle \delta\mathbf{a}^\dagger\delta\mathbf{a}\rangle & \langle \delta\mathbf{a}^\dagger\delta\mathbf{a}^\dagger\rangle \end{pmatrix}\end{aligned} \tag{S12}$$

The correlation matrix C which multiplies the gradients v is constructed from the statistics of the initial field, which allows for straightforward inclusion of the effects of excess noise, multimode correlations (e.g., entanglement), and phase-sensitive correlations (e.g., from squeezed states of light). This reformulation shows that the noise is in fact largely understood in terms of the gradient of the classical transformation of the light with respect to the initial conditions. This enables one to predict and understand the dynamics of fluctuations and noise based on the classical understanding of nonlinear optical effects developed over recent years. That said, one key aspect of this framework that goes "beyond" many classical studies is that: in this framework, one must also study the sensitivity of the system to dark modes, as it is these which often limits the noise performance in the presence of interactions.

S1.1. Application to shot-noise limited inputs

In this section, we present a simple limiting case of Eq. S12 which provides a versatile formula for understanding quantum noise in systems with many-degrees of freedom. This special case will also more explicitly illustrate the physics of the quantum optical law of total variance.

We consider the limiting case in which the initial inputs have only vacuum noise (e.g. no excess noise and no phase-sensitive correlations). Cases with excess noise are treated later on in the Supplement. In that case, the only non-vanishing elements of the correlation matrix C are $\langle \delta a_i \delta a_j^\dagger\rangle = \delta_{ij}$. In that case, we may immediately write that the variance of any observable X is given as

$$(\Delta X)^2 = (\partial X/\partial \boldsymbol{\alpha})^\dagger(\partial X/\partial \boldsymbol{\alpha}) = \sum_k \left|\frac{\partial X}{\partial \alpha_k}\right|^2. \tag{S13}$$

In writing this, we have used the fact that X must be real-valued (as it is an observable), implying $\partial X/\partial \alpha_k^* = (\partial X/\partial \alpha_k)^*$ for any mode k.

This expression tells us that to understand the noise in X, we can ask how sensitive it is classically to a change in the initial conditions in mode k, where the typical magnitude of such a change is of order 1. Note that the α_k are in "photon units", so that $|\alpha|^2$ represents a number of photons. So, for a macroscopic initial occupation of some mode with n photons on average, $|\alpha| \sim \sqrt{n}$. If some observable is highly sensitive to changes of initial conditions in some mode, or even moderately sensitive but to many modes, one can expect X to be noisy. This is to say that observables which feature a high degree of connectivity will display also large noise. This can be understood from the fact that all of the contributions to the noise in X are non-negative. Importantly, since the contributions to the noise are all non-negative, the only way to have zero noise in the output is if all the derivatives vanish. This can be understood as a more general version of a phenomenon called *partition noise*, in which the splitting of some quantity into orthogonal channels leads to enhanced noise for those individual channels. Our framework not only makes these effects evident, but explains what happens when an in-principle infinite or continuous number of modes is involved. Of course, our framework can also deal with a wide variety of noise phenomena in addition to partition noise effects, as we will illustrate through examples in Section 2.

S1.2. Case of an input-output theory specified by position and momenta

In the derivation above, we considered an input-output theory specified in terms of complex amplitudes α, α^*. That said, the key physical idea, of looking at small variations of classical inputs, is general, and holds for other types of degrees of freedom a system might have. For example, if instead of a wave system, we have a system of particles whose degrees of freedom are position and momenta, then we can calculate noise by differentiating with respect to position and momenta. In what follows, we derive the quantum mechanical law of total variance for the case of a single particle described by a position and momentum. The multi-particle generalization, as well as the generalization to systems with particle and wave degrees of freedom, is straightforward.

To derive the case of a position-momentum description, the simplest thing to do is to take the quantum mechanical law of total variance for a system described by complex amplitudes α, α^*, and make a variable transformation to position and momentum variables Q, P (one can convince themselves a particle system can be described by complex degrees of freedom α, α^* that are simple linear combinations of Q, P). Defining: $Q = (\alpha + \alpha^*)/2$ and $P = (\alpha - \alpha^*)/2i$, and using the

chain rule to relate α, α^* derivatives to Q, P derivatives, one immediately gets:

$$(\Delta X)^2 = v^T C v, \text{where}$$
$$v = \left(\frac{\partial X}{\partial Q}\ \frac{\partial X}{\partial P}\right)^T$$
$$C = \begin{pmatrix} \langle(\delta Q)^2\rangle & \frac{1}{2}\langle\delta Q\delta P + \delta P\delta Q\rangle \\ \langle\frac{1}{2}\langle\delta Q\delta P + \delta P\delta Q\rangle & \langle(\delta P)^2\rangle \end{pmatrix}. \quad \text{(S14)}$$

The generalization to multiple positions and momenta follows immediately.

S2. APPLYING THE FRAMEWORK TO TEXTBOOK PROBLEMS IN QUANTUM OPTICS

In this section, we apply the framework to a few cases where the answers are widely known. We do this so that the interested reader can work through some simple analytical examples, convince themselves of the framework, and also see the variety of cases that can be dealt with. In some cases, it will be the case that this version of the derivation allows one to do the calculations more simply than the standard textbook derivation. It also enables a new decomposition, allowing identification of how different noise sources contribute.

S2.1. Quantum noise in linear dissipation

Consider a linear dissipative process which passes through a fraction η^2 of incident light. A consistent model of the linear dissipation treats the attenuation as a beamsplitting operation which transfers the attenuated energy to a second mode. Calling the input modes α, β, and denoting the outputs by α', β', we have

$$\alpha' = -\eta\alpha + i\sqrt{1-\eta^2}\beta$$
$$\beta' = i\sqrt{1-\eta^2}\alpha + \eta\beta. \quad \text{(S15)}$$

Now, let's consider the transformation of an operator such as the quadrature operator

$$X' = \alpha' e^{i\theta} + \alpha'^* e^{-i\theta}, \quad \text{(S16)}$$

and evaluate the variance $(\Delta X)^2$, which probes for example quadrature squeezing. Using the derivatives $\partial X'/\partial\alpha == -re^{i\theta}, \partial X'/\partial\alpha^* = -re^{-i\theta}, \partial X'/\partial\beta = ite^{i\theta}, \partial X'/\partial\beta^* = -ite^{-i\theta}$, we

have

$$
\begin{aligned}
(\Delta X)^2 &= v^T C v, \text{where} \\
v &= (\partial X/\partial\alpha \; \partial X/\partial\beta \; \partial X/\partial\alpha^* \; \partial X/\partial\beta^*)^T \\
C &= \begin{pmatrix} \langle\delta\alpha\delta\alpha\rangle & \langle\delta\alpha\delta\beta\rangle & \langle\delta\alpha\delta\alpha^*\rangle & \langle\delta\alpha\delta\beta^*\rangle \\ \langle\delta\beta\delta\alpha\rangle & \langle\delta\beta\delta\beta\rangle & \langle\delta\beta\delta\alpha^*\rangle & \langle\delta\beta\delta\beta^*\rangle \\ \langle\delta\alpha^*\delta\alpha\rangle & \langle\delta\alpha^*\delta\beta\rangle & \langle\delta\alpha^*\delta\alpha^*\rangle & \langle\delta\alpha^*\delta\beta^*\rangle \\ \langle\delta\beta^*\delta\alpha\rangle & \langle\delta\beta^*\delta\beta\rangle & \langle\delta\beta^*\delta\alpha^*\rangle & \langle\delta\beta^*\delta\beta^*\rangle \end{pmatrix}.
\end{aligned}
\tag{S17}
$$

This can easily be shown to match the quantum calculation, using the operator transformations $a' = -\eta a + i\sqrt{1-\eta^2}b$, $b' = i\sqrt{1-\eta^2}a - \eta b$.

As a more explicit example, let us also consider the change in the photon number variance after propagation through a linear beamsplitter. The output intensity in for example, mode 1, is given as

$$
n' = \alpha'^*\alpha' = (-\eta\alpha^* - i\sqrt{1-\eta^2}\beta^*)(\eta\alpha + i\sqrt{1-\eta^2}\beta). \tag{S18}
$$

Let us consider the simple, but very common case, in which the input light in mode a doesn't have phase-sensitive correlations, and the light in mode b has no photons incident. Then, the output fluctuations, according to this formalism are

$$
(\Delta n')^2 = \left|\frac{\partial n'}{\partial\alpha}\right|^2 \langle\delta\alpha\delta\alpha^* + \delta\alpha^*\delta\alpha\rangle + \left|\frac{\partial n'}{\partial\beta}\right|^2 \tag{S19}
$$

Using $\partial n'/\partial\alpha = \eta^2\alpha^*$ and $\partial n'/\partial\beta = -i\eta\sqrt{1-\eta^2}\alpha^*$ (note that β is zero so the quadratic term doesn't contribute), we get

$$
\begin{aligned}
(\Delta n')^2 &= \eta^4|\alpha|^2\langle\delta\alpha\delta\alpha^* + \delta\alpha^*\delta\alpha\rangle + \eta^2(1-\eta^2)|\alpha|^2 \\
&\equiv l^2(\Delta n)^2 + l(1-l)\langle n\rangle,
\end{aligned}
\tag{S20}
$$

where we have defined the transmission fraction $l = \eta^2$. This result is equivalent to the well-known formula which describes the change in intensity statistics due to dissipation (in more technical terms, this formula prescribes the noise figure of linear dissipation) [1].

S2.2. Continuous dissipation

In this section, we show that the framework also accounts for continuous processes of dissipation, where vacuum fluctuations are continuously coupled to the system. This example represents

a significant extension in the scope of our framework. From the quantum optical law of total variance, we see that the noise depends on derivatives with respect to initial conditions. However, the equation of motion for a leaky cavity leads to vacuum fluctuations which can enter the cavity continuously, not just at the initial time, raising questions as to whether our framework could capture such cases. However, in the quantum mechanical theory of dissipation, it is known that these terms which describe continuously entering vacuum fluctuations (specifically, the term is a Langevin force) can be expressed in terms of the initial conditions of a reservoir of far-field modes. Therefore, the effect of continuous loss is treatable in our framework. We now demonstrate this.

Let us consider the classical description of a leaky cavity within temporal coupled mode theory [2]. For a cavity with one in-coupling port, we can write

$$\dot{a} = -\kappa a + \sqrt{2\kappa}b(t), \tag{S21}$$

where κ is the rate of decay of the intracavity amplitude. The field $a(t)$ is normalized such that $|a(t)|^2$ is the number of photons in the cavity. The term $b(t)$ is an injected input signal, normalized so that $|b(t)|^2$ is the number of photons per unit time incident on the cavity. For a cavity with no input, the term $b(t)$ is zero. However, in quantum calculations related to noise, we need to keep it: the essence of the quantum theory of noise presented in this work is that by treating $b(t)$ as a vacuum-fluctuating amplitude, the noise can be calculated by looking at derivatives with respect to the initial conditions. Said differently, while the field $b(t)$ might be zero on average, the derivative of the cavity field with respect to b is not zero. With that said, we calculate the evolution of the photon number noise with respect to time, due to cavity leakage.

The observable of interest is $a^*(t)a(t)$. The field $a(t)$ follows from Eq. S21 as

$$a(t) = a(0)e^{-\kappa t} + \sqrt{2\kappa}\int_0^t dt' e^{-k(t-t')}b(t'). \tag{S22}$$

The field $b(t)$ can be written in terms of independent degrees of freedom (which we will differentiate with respect to) as

$$b(t) = \int \frac{d\omega}{2\pi} e^{-i\omega t} b(\omega). \tag{S23}$$

Plugging this in to the solution for $a(t)$, we have

$$a(t) = a(0)e^{-\kappa t} + \sqrt{2\kappa}\int \frac{d\omega}{2\pi} b(\omega)\left[\frac{e^{-i\omega t} - e^{-\kappa t}}{\kappa - i\omega}\right]. \tag{S24}$$

The photon number is given by $n(t) = a^*(t)a(t)$ For an initial cavity state with excess phase-insensitive noise described by a Fano factor F, application of the quantum optical law of total variance yields

$$(\Delta n(t))^2 = \left|\frac{\partial n(t)}{\partial a(0)}\right|^2 F + \int \frac{d\omega}{2\pi}\left|\frac{\partial n(t)}{\partial b(\omega)}\right|^2. \tag{S25}$$

Recalling that the derivatives are taken with respect to the initial conditions $a(0) \neq 0$ and $b(\omega) = 0$, we have that

$$\left|\frac{\partial n(t)}{\partial a(0)}\right|^2 = e^{-4\kappa t} n(0) \tag{S26}$$

and

$$\left|\frac{\partial n(t)}{\partial b(\omega)}\right|^2 = e^{-2\kappa t} n(0)\frac{2\kappa}{\kappa^2+\omega^2}(1 + e^{-2\kappa t} - 2\text{Re}\, e^{-i\omega t-\kappa t}). \tag{S27}$$

Evaluating the frequency integral in the quantum optical law of total variance yields

$$(\Delta n(t))^2 = l^2(\Delta n(0))^2 + l(1-l)\bar{n}(0), \tag{S28}$$

where $l = e^{-2\kappa t}$ is the fractional intensity loss. This is precisely what is given from a quantum optical calculation.

S2.3. Parametric amplification

Here, we show how this framework readily allows one to consider parametric gain such as that provided by optical parametric amplifiers and oscillators which lead to squeezing. This shows immediately that our framework applies to second-order nonlinear effects.

For simplicity, we'll consider the case of a degenerate parametric amplifier: the non-degenerate case can also readily be considered. The equations of motion are [3]

$$\begin{aligned} \dot{a} &= \Omega a^* \\ \dot{a}^* &= \Omega^* a. \end{aligned} \tag{S29}$$

The solution is

$$\begin{pmatrix} a(t) \\ a^*(t) \end{pmatrix} = \exp\left[\begin{pmatrix} 0 & \Omega t \\ \Omega^* t & 0 \end{pmatrix}\right]\begin{pmatrix} a(0) \\ a^*(0) \end{pmatrix} = \begin{pmatrix} \cosh(|\Omega| t)\, a(0) + \sinh(|\Omega| t) e^{i\phi} a^*(0) \\ \sinh(|\Omega| t) e^{-i\phi} a(0) + \cosh(|\Omega| t)\, a^*(0) \end{pmatrix} \tag{S30}$$

where we have defined $\Omega = |\Omega| e^{i\phi}$. Let us now consider a quadrature operator $X_\varphi = a e^{i\varphi} + a^\dagger e^{-i\varphi}$. With this normalization, the quadrature variance in the vacuum state is one. Let us now ask about

the squeezing of an initially injected vacuum state. From the quantum optical law of total variance, the quadrature variance is simply:

$$(\Delta X_\varphi)^2 = \Big|\frac{\partial X_\varphi(t)}{\partial a(0)}\Big|^2 = \Big|\cosh\left(|\Omega|t\right)e^{i\varphi} + \sinh(|\Omega|t)e^{-i\phi}e^{-i\varphi}\Big|^2. \tag{S31}$$

We briefly state a few limits of this formula. $\Omega = 0$ trivially returns $(\Delta X_\varphi)^2 = 1$. For a real pump phase, $\phi = 0$, we see that the amplitude quadrature variance ($\varphi = 0$) becomes $(\Delta X_0)^2 = e^{2\Omega t}$, while the phase quadrature variance becomes $(\Delta X_{\pi/2})^2 = e^{-2\Omega t}$. This is precisely the expected behavior.

Let us highlight an important extension of the formula which is sigificantly more involved in the standard quantum derivation: the effect of excess noise in the mode being amplified. We'll show the case of phase-insensitive noise, but the phase-sensitive case readily derived as well. The influence of excess phase-sensitve noise is to have non-zero expectation values $\langle \delta a\delta a^\dagger + \delta a^\dagger \delta a\rangle$. This expectation value can readily be shown to be the Fano factor, F, for the incident light (the ratio of the intensity fluctuations to the mean intensity: for shot-noise limited light $F = 1$, while for sub- (super-) Poissonian light, $F < (>)1$. In this case, the quantum optical law of total variance immediately implies

$$(\Delta X_\varphi)^2 = F\Big|\frac{\partial X_\varphi(t)}{\partial a(0)}\Big|^2 = F\Big|\cosh\left(|\Omega|t\right)e^{i\varphi} + \sinh(|\Omega|t)e^{-i\phi}e^{-i\varphi}\Big|^2. \tag{S32}$$

S2.4. Linear amplifier

In this section, we show that a linear amplifier can also be readily described with this framework. We will make use of the result derived in the section on continuous linear dissipation. In fact, it turns out that: by taking $-\kappa \to G$ in the first term, and $\sqrt{2\kappa} \to \sqrt{2G}$ in the second term, one may repeat the derivation, arriving at the result that the intensity fluctuations of the output are

$$(\Delta n)^2 = g^2(\Delta n(0))^2 + g(g-1)\bar{n}(0), \tag{S33}$$

with $g = e^{2Gt}$, which agrees with the result in textbooks, e.g. [3]. In the shot-noise limited case, this leads to intensity fluctuations $g\bar{n}(0)(2g-1)$.

S2.5. Kerr squeezing

In this section, we show how to apply our theory to calculate quadrature squeezing in the Kerr effect. The standard framework derivation is presented in [4]. The equation of motion of a single

mode of light undergoing self-phase modulation is

$$\dot{\alpha} = iK|\alpha|^2\alpha, \tag{S34}$$

whose solution is

$$\alpha(t) = e^{i\theta\alpha^*\alpha}\alpha, \tag{S35}$$

where $\alpha = \alpha(0)$ and $\theta = Kt$ is the nonlinear phase shift per photon. Evidently then, the intensity fluctuations follow simply as

$$(\Delta n)^2 = \left|\frac{\partial n}{\partial \alpha}\right|^2 = |\alpha|^2, \tag{S36}$$

where we have used $|\alpha(t)|^2 = |\alpha|^2$. More interesting are the fluctuations of the quadrature: $X_\varphi = \alpha(t)e^{i\varphi} + \alpha^*(t)e^{-i\varphi}$. This follows immediately from our theory as

$$(\Delta X_\varphi)^2 = \left|\frac{\partial X_\varphi}{\partial \alpha}\right|^2 = \left|(1 + i\theta|\alpha|^2)e^{2i\varphi+2i\theta|\alpha|^2} - i\theta\alpha^{*2}\right|^2. \tag{S37}$$

Related to this is the use of a Sagnac interferometer to produce squeezed vacuum using the Kerr effect. Without an interferometer, the pure Kerr effect leads to a bright (coherent) squeezed state. Since squeezed vacuum states are important for applications such as gravitational-wave interferometry, techniques to convert bright squeezed states into squeezed vacuum are important. The way to do this is to send light into a 50/50 Sagnac interferometer. Out of one port will come squeezed vacuum. The quadrature squeezing in that case can be readily derived. Passing the light through a 50/50 beamsplitter, followed by the Kerr effect, followed by another 50/50, leads to the following output field:

$$\alpha'(\alpha, \alpha^*, \beta, \beta^*) = -\frac{1}{2}e^{\frac{1}{2}i\theta(-\alpha^*-i\beta^*)(-\alpha+i\beta)}(-\alpha + i\beta) + \frac{i}{2}e^{\frac{1}{2}i\theta(-i\alpha^*-\beta^*)(i\alpha-\beta)}(i\alpha - \beta). \tag{S38}$$

The output noise is then

$$(\Delta X_\varphi)^2 = \left|\frac{\partial X_\varphi}{\partial \alpha}\right|^2 + \left|\frac{\partial X_\varphi}{\partial \beta}\right|^2 = \left|(1 + \frac{1}{2}i\theta|\alpha|^2)e^{i(2\varphi+\theta|\alpha|^2)} - \frac{1}{2}i\theta\alpha^{*2}\right|^2, \tag{S39}$$

which, one can show that the maximum squeezing is $4\Phi^2$, where Φ is the nonlinear phase shift of the light in the Kerr medium (it is half the phase-shift accumulated by the input, since the input gets split in half). Also notice that the squeezing is simply that of the fiber alone (with no beamsplitters), but evaluated at half the intensity (which makes sense since each nonlinear arm has

half the input intensity after going through a 50/50 splitter).

---

[1] Hans-A Bachor and Timothy C Ralph. *A guide to experiments in quantum optics*. John Wiley & Sons, 2019.

[2] Hermann A Haus. Waves and fields in optoelectronics. 1984.

[3] Marlan O Scully and M Suhail Zubairy. Quantum optics, 1999.

[4] Hermann A Haus. *Electromagnetic noise and quantum optical measurements*. Springer Science & Business Media, 2000.
